# Surface-Shaving Proteomics of Mycobacterium marinum Identifies Biofilm Subtype-Specific Changes Affecting Virulence, Tolerance, and Persistence

**DOI:** 10.1128/mSystems.00500-21

**Published:** 2021-06-22

**Authors:** Kirsi Savijoki, Henna Myllymäki, Hanna Luukinen, Lauri Paulamäki, Leena-Maija Vanha-aho, Aleksandra Svorjova, Ilkka Miettinen, Adyary Fallarero, Teemu O. Ihalainen, Jari Yli-Kauhaluoma, Tuula A. Nyman, Mataleena Parikka

**Affiliations:** a Drug Research Program, Division of Pharmaceutical Biosciences, Faculty of Pharmacy, University of Helsinki, Helsinki, Finland; b Faculty of Medicine and Health Technology, Tampere University, Tampere, Finland; c Drug Research Program, Division of Pharmaceutical Chemistry and Technology, Faculty of Pharmacy, University of Helsinki, Helsinki, Finland; d Institute of Clinical Medicine, Department of Immunology, Rikshospitalet, Oslo University Hospital, Oslo, Norway; California State University, Fresno

**Keywords:** biofilm matrix, biofilms, cell surface proteomics, *Mycobacterium marinum*, persistence, tolerance

## Abstract

The complex cell wall and biofilm matrix (ECM) act as key barriers to antibiotics in mycobacteria. Here, the ECM and envelope proteins of Mycobacterium marinum ATCC 927, a nontuberculous mycobacterial model, were monitored over 3 months by label-free proteomics and compared with cell surface proteins on planktonic cells to uncover pathways leading to virulence, tolerance, and persistence. We show that ATCC 927 forms pellicle-type and submerged-type biofilms (PBFs and SBFs, respectively) after 2 weeks and 2 days of growth, respectively, and that the increased CelA1 synthesis in this strain prevents biofilm formation and leads to reduced rifampicin tolerance. The proteomic data suggest that specific changes in mycolic acid synthesis (cord factor), Esx1 secretion, and cell wall adhesins explain the appearance of PBFs as ribbon-like cords and SBFs as lichen-like structures. A subpopulation of cells resisting 64× MIC rifampicin (persisters) was detected in both biofilm subtypes and already in 1-week-old SBFs. The key forces boosting their development could include subtype-dependent changes in asymmetric cell division, cell wall biogenesis, tricarboxylic acid/glyoxylate cycle activities, and energy/redox/iron metabolisms. The effect of various ambient oxygen tensions on each cell type and nonclassical protein secretion are likely factors explaining the majority of the subtype-specific changes. The proteomic findings also imply that Esx1-type protein secretion is more efficient in planktonic (PL) and PBF cells, while SBF may prefer both the Esx5 and nonclassical pathways to control virulence and prolonged viability/persistence. In conclusion, this study reports the first proteomic insight into aging mycobacterial biofilm ECMs and indicates biofilm subtype-dependent mechanisms conferring increased adaptive potential and virulence of nontuberculous mycobacteria.

**IMPORTANCE** Mycobacteria are naturally resilient, and mycobacterial infections are notoriously difficult to treat with antibiotics, with biofilm formation being the main factor complicating the successful treatment of tuberculosis (TB). The present study shows that nontuberculous Mycobacterium marinum ATCC 927 forms submerged- and pellicle-type biofilms with lichen- and ribbon-like structures, respectively, as well as persister cells under the same conditions. We show that both biofilm subtypes differ in terms of virulence-, tolerance-, and persistence-conferring activities, highlighting the fact that both subtypes should be targeted to maximize the power of antimycobacterial treatment therapies.

## INTRODUCTION

Tuberculosis (TB) remains a major global health issue, with approximately 10 million new cases and 1.4 million deaths in 2019 (1). The causative agent, Mycobacterium tuberculosis (Mtb), is carried by an estimated one-quarter of the human population as a latent infection, which has a 5% to 10% lifetime risk of developing into TB disease. In addition, the emergence of drug-resistant Mtb strains continues to be a public health threat, with approximately half a million new cases in 2019. Even in the case of drug-sensitive Mtb strains, the first-line antibiotic treatment requires the use of four antimicrobials over a course of at least 6 months (WHO, 2020). Moreover, despite successful treatment, the recurrence of TB carries a substantial risk, especially among immunocompromised patients (2, 3). The heterogeneity of the standard treatment outcome is also evident in positron emission tomography-computed tomography (PET-CT) images showing nonresolving and active lesions and the presence of Mtb mRNA in sputum samples. This suggests that a significant proportion of patients generate viable mycobacteria in their lungs even after clinically curative antibiotic treatment (4). In a rabbit TB model, it was further shown that the caseum of granulomas contains Mtb that is highly tolerant to most anti-TB drugs (5). The complex mycobacterial cell wall, involving capsule and outer/inner membranes connected by a dense mycolyl-arabinogalactan-peptidoglycan with high lipid levels, is the main barrier that protects the bacterial cells against drugs (6). While the mechanisms leading to drug tolerance in TB have remained poorly understood, biofilm formation was recently indicated as one of the strategies to increase viability, tolerance, and persistence (7–10).

Biofilm formation is defined as adherent growth within self-produced extracellular matrix (ECM) consisting of proteins, polysaccharides, and DNA/RNA, and it is the strategy bacteria use to escape the effects of antibiotics and host defense systems (11–13). Mycobacteria use phenotypically distinct biofilm subtypes for growth, which physiologically differ from planktonic-type growth. These include (i) floating/pellicle-type biofilms (PBFs) at the air-liquid interface having an ECM rich in free mycolic acids (MAs) and with a frequent cord/ribbon-like appearance, while (ii) submerged-type biofilms (SBFs) show adherent growth on a solid substratum (11, 14–16). The capsule layer plays a vital role in triggering biofilm growth in mycobacteria, as Tween 80 (nonionic surfactant) has been shown to detach the capsule layer and prevent biofilm formation of culture cells (17). Thus, this labile layer forming the first molecular interaction with the host/environment is likely to involve key factors contributing to persistence/adaptation and search of anti-TB targets. Although several studies on mycobacteria have pinpointed cellular pathways and proteins that affect the capsule/cell wall and biofilm formation (9, 14, 17–25), systematic investigation of the factors that directly interact with the surrounding environment is necessary to be able to maximize the power of antimycobacterial treatment therapies.

Mycobacterium marinum (Mmr) has proven to be an excellent alternative model pathogen for slow-growing Mtb, as it allows for the investigation of TB-like chronic and latent infections in its natural host, the zebrafish (26–29). Cultured mycobacterial biofilms have been used to understand resilient bacterial phenotypes emerging in mycobacterial infections. However, the distinct phenotypic profiles associated with PBFs and SBFs, including marker proteins discriminating the two biofilm subtypes, have remained poorly understood. To shed light on the specific attributes linking these biologically different biofilm subtypes to their phenotypes, we first cultured Mmr strain ATCC 927 to create *in vitro* biofilms. These biofilms were then imaged using widefield deconvolution microscopy (WDeM) to investigate temporal effects on biofilm architectures. Label-free quantitative (LFQ) proteomics was next used to uncover the ECM proteome dynamics in maturing Mmr biofilms and to identify the cell surface proteins (proteome) on Mmr cells grown in Tween 80, a detergent known to prevent cells from clumping and forming a biofilm (17). The key proteome findings were validated by gene overexpression studies to indicate cellulose-dependent biofilm formation as well as by biofilm killing assays to confirm the formation of persister cells in both biofilm subtypes. To the best of our knowledge, this is the first study monitoring mycobacterial ECM proteomes over 3 months’ time as well as protein and morphological phenotypic markers for distinguishing defined biofilm subtypes.

## RESULTS

### SBFs and PBFs show distinct morphological characteristics.

**FIG 1:**
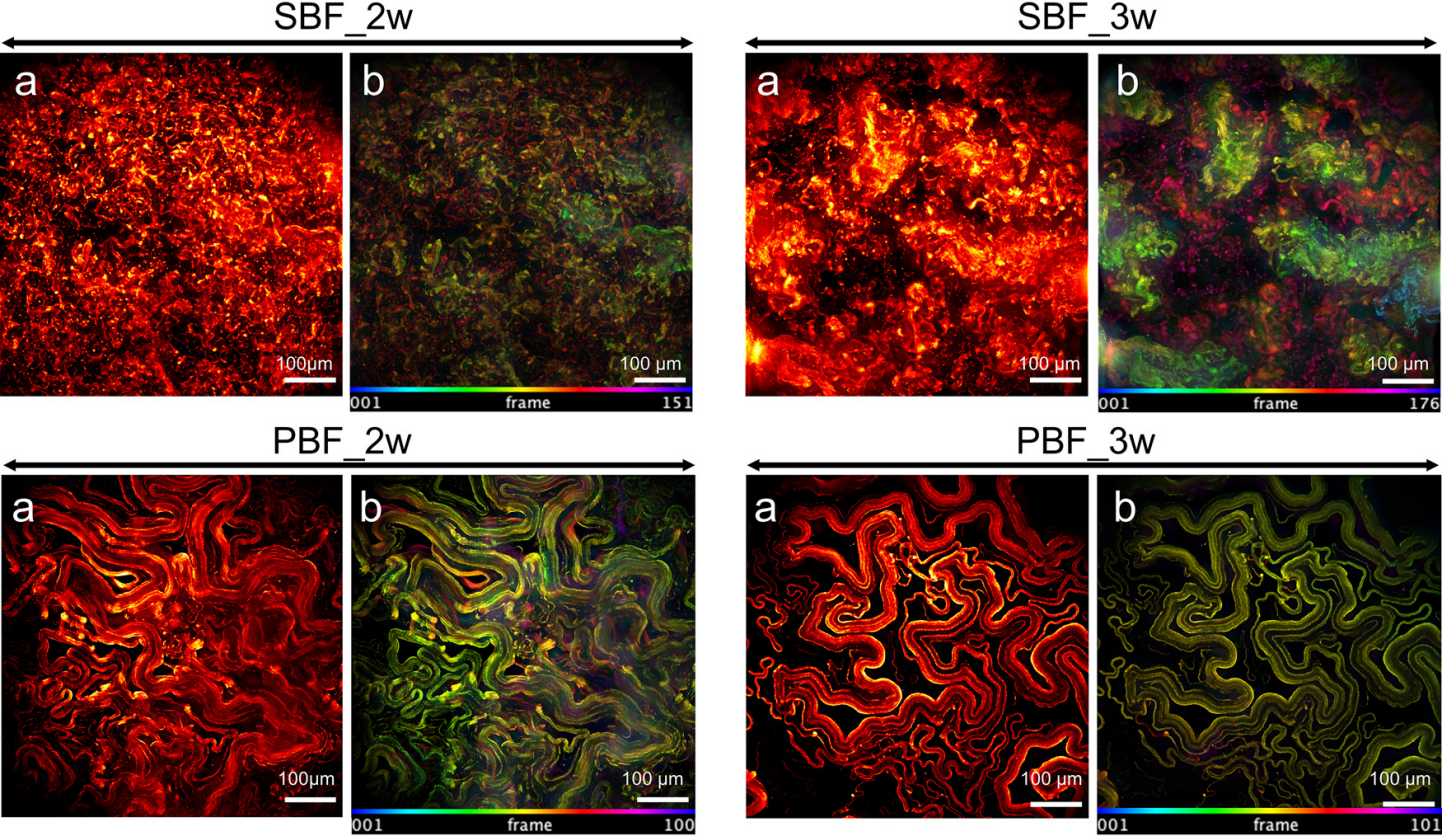
Mmr biofilms show distinct growth morphologies after 2-weeks of growth. SBFs grow with lichen-like structures, whereas PBFs have a ribbon-like cords morphology, which becomes more defined with maturation (after 3 weeks). The WDeM images are maximum-intensity projections of 2- and 3-week-old biofilms (a) together with an image where the *z* position is color coded (b); frame interval is 2 μm.

The kinetics of development and maturation as well as the morphology of mycobacterial PBFs and SBFs have been reported to differ substantially (8). Here, we first show that that Mmr forms PBFs at the air-liquid interphase and that SBFs attached to the bottom of the culture well under the same physiological *in vitro* conditions after 2 weeks of growth (see Fig. S1A in the supplemental material). The SBF subtype developed earlier (visible already after 2 days of culture) than the PBF, which was not clearly distinguishable before 2 weeks of growth. Next, we investigated the three-dimensional morphology of Mmr biofilms in more detail by culturing Mmr cells carrying the pTEC27 plasmid with the tdTomato fluorescent marker gene (29) for 2 and 3 weeks to produce PBFs and SBFs and analyzing the biofilms by widefield deconvolution microscopy (WDeM). Figure 1 shows that Mmr forms organized three-dimensional structures with distinctive subtype-specific morphological features. For the SBF, the structures displayed a lichen- or moss-like appearance, having tens-of-microns-high feature structures rising from the biofilm base after 2 weeks (Fig. 1, top). In comparison, the morphology of the PBF subtype was very different by the first time point, showing flat ribbon-like structures without any protruding structures (Fig. 1, bottom). Defined, extensive structures in all dimensions, although less dense than those detected at the 2-week time point, were found for both biofilm subtypes also after 3 weeks of growth.

10.1128/mSystems.00500-21.1 FIG S1 (A) Pellicle-type (PBF) and submerged (SBF) biofilms after culturing for 2 weeks (left) and 12 weeks (right). (B) Distribution of overlapping protein identifications within four replica samples from planktonic cell surfaces and biofilm ECMs. PL_log, logarithmic planktonic cells; SBF, submerged-type biofilms; PBF, pellicle-type biofilms. Download FIG S1, PDF file, 0.05 MB. Copyright © 2021 Savijoki et al. 2021 Savijoki et al. https://creativecommons.org/licenses/by/4.0/ This content is distributed under the terms of the Creative Commons Attribution 4.0 International license.

### Submerged biofilms exhibit the greatest ECM proteome diversity.

**FIG 2:**
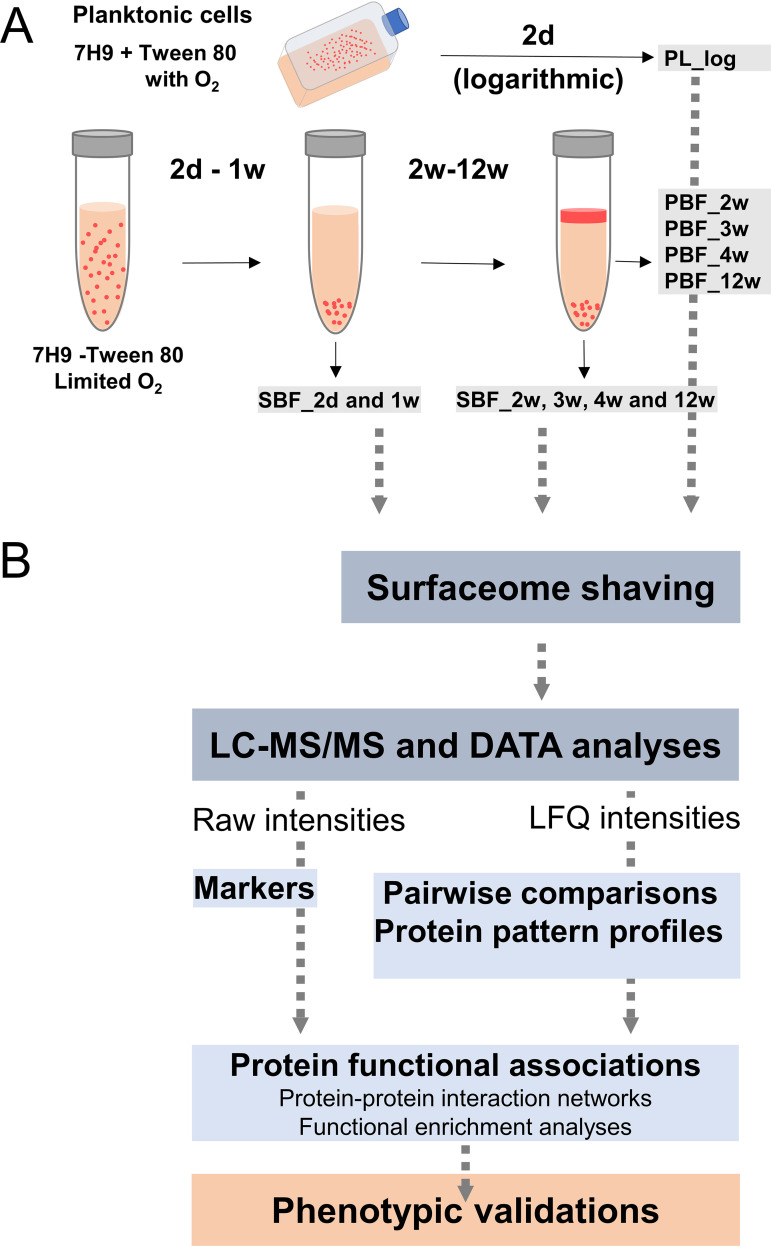
(A) Workflow depicting the conditions and time points used for preparing the planktonic and biofilm cells of Mmr. Gray arrows indicate sampling time points for pellicle (PBF) and submerged (SBF) biofilms. (B) Workflow used for identification of surface proteins associated with planktonic (PL_log) cells, PBFs, and SBFs. Marker proteins were identified by comparing the raw intensity data, statistically significant protein abundance changes by pairwise comparisons of the log_2_-converted LFQ data, and the protein coabundance patterns by subjecting the LFQ data to imputation and Z-score normalization. STRING and pathway enrichment analyses were conducted on the selected heat map clusters and necessary phenotypic assays to validate the key proteome differences.

As the phenotypic profiles of PBFs and SBFs are clearly different, their ECM proteomes were next quantitatively monitored and compared during the development and maturation stages. To this end, the PBF and SBF cells at the points shown in Fig. 2A were subjected to trypsin/Lys-C digestion as well as liquid chromatography-tandem mass spectrometry (LC-MS/MS)-based protein identification and LFQ proteomics (all data available via PRIDE with identifier PXD02010). Logarithmic state planktonic cells (PL_log), representing single-cell cultures, were obtained by growing the Mmr strain in the presence of Tween 80. The quality of each data set was high: 84.7% or all proteins were identified with at least three or more matching peptides, with an average sequence coverage of approximately 31%, and only 11% of proteins were categorized as single-peptide hits. In addition, a broad overlap in protein identifications was detected within the four biological replica samples; 41% to 89% of the proteins were shared by each replicate, with the 2-week PBF and the 3-week SBF showing the highest variation between replicates (Fig. S1B). Table S1 lists the proteins detected in at least two of four replica samples. An outlier replicate associated with one of the SBF identification replica sets at the 3-week time point was excluded from subsequent data analyses. The numbers of detected proteins were 1,132, 1,957, and 2,133 for the PL, PBF, and SBF cells, respectively.

10.1128/mSystems.00500-21.4 TABLE S1 List of all proteins identified on Mmr cells during different growth modes. The colored cells refer to the average (log_2_) raw intensity values for proteins detected in at least two replica samples. Cells in grey, protein was not detected; PL_log, logarithmic planktonic cells; PBF, pellicle-type biofilm cells; SBF, submerged-type biofilm cells. Download Table S1, XLSX file, 2.0 MB. Copyright © 2021 Savijoki et al. 2021 Savijoki et al. https://creativecommons.org/licenses/by/4.0/ This content is distributed under the terms of the Creative Commons Attribution 4.0 International license.

### Cytoplasmic protein export/release is most efficient in submerged biofilms.

Figure S2A shows the distributions of all identified proteins according to their predicted secretion motif (Sec/SPII, TatP/SPI, LIPO/SPII, and type VII secretion [T7SS]; SecretomeP) and the number of transmembrane spanning domains (TMDs). The most notable differences were detected for membrane proteins with six to ten TMDs as well as in the number of cytoplasmic proteins. Nearly 2-fold more trehalose dimycolate (TDM) proteins were detected from the PL cells than from the biofilm cells. In contrast, 2-fold more cytoplasmic proteins predicted to be exported out of the cells via a nonclassical route (SecretomeP) were identified from the biofilm ECMs (*n*, 300) in comparison to that from the PL cells (*n*, 150). For many of these proteins, a secondary function as a moonlighting protein (30) was indicated (Table S1). In addition, more than 900, 1,600, and 1,800 cytoplasmic proteins identified in the PL, PBF, and SBF cells, respectively, contained no motifs for classical or nonclassical secretion and were assigned here as “others” (Table S1).

10.1128/mSystems.00500-21.2 FIG S2 (A) Distribution of identified proteins in terms of their predicted secretion motifs and the number of predicted TMDs. PL_log, logarithmic planktonic cells; PBF_all and SBF_all, all identified proteins from pellicle and submerged biofilm matrices, respectively; “other,” proteins without any known motifs for classical or nonclassical secretion. (B) Venn diagrams indicating the core and marker proteomes within all identifications (planktonic and biofilms) at each time point. Download FIG S2, PDF file, 0.05 MB. Copyright © 2021 Savijoki et al. 2021 Savijoki et al. https://creativecommons.org/licenses/by/4.0/ This content is distributed under the terms of the Creative Commons Attribution 4.0 International license.

### Most significant protein abundance changes specific to planktonic and biofilm cells.

The Venn diagram in Fig. S2B indicates the highest number of specifically identified proteins in the SBFs (*n*, 173) and the lowest in the PBFs (*n*, 16), while no unique identifications were detected for the PL cells. The uniquely detected proteins with the highest raw intensity values included a signal transduction-associated serine/threonine-protein kinase (PknL), an LGFP-repeat protein specific to SBFs, and a β-1,3-endoglucanase and bacterioferritin BfrA specific to PBFs (see Table S2). The proteins detected with the highest intensity values and only in the biofilm ECMs included an error-prone polymerase DinB, a preprotein sec-translocase subunit YajC, a cytochrome P-450 monooxygenase, a PE family immunogen, and a signal transduction-related adenylate cyclase involved in cyclic di-AMP biosynthesis (Table S2).

10.1128/mSystems.00500-21.5 TABLE S2 Proteins specific to pellicle biofilms (PBFs), submerged biofilms (SBFs), and to both biofilms that lack an identifiable counterpart on planktonic cell surfaces. Proteins detected in each replica samples are shown. Color gradient bar refers to log2-transformed raw intensity values (average, *n* ≥ 2): blue, low abundance; yellow, high abundance; PBF, pellicle biofilm; SBF, submerged biofilm. Download Table S2, XLSX file, 0.3 MB. Copyright © 2021 Savijoki et al. 2021 Savijoki et al. https://creativecommons.org/licenses/by/4.0/ This content is distributed under the terms of the Creative Commons Attribution 4.0 International license.

**FIG 3:**
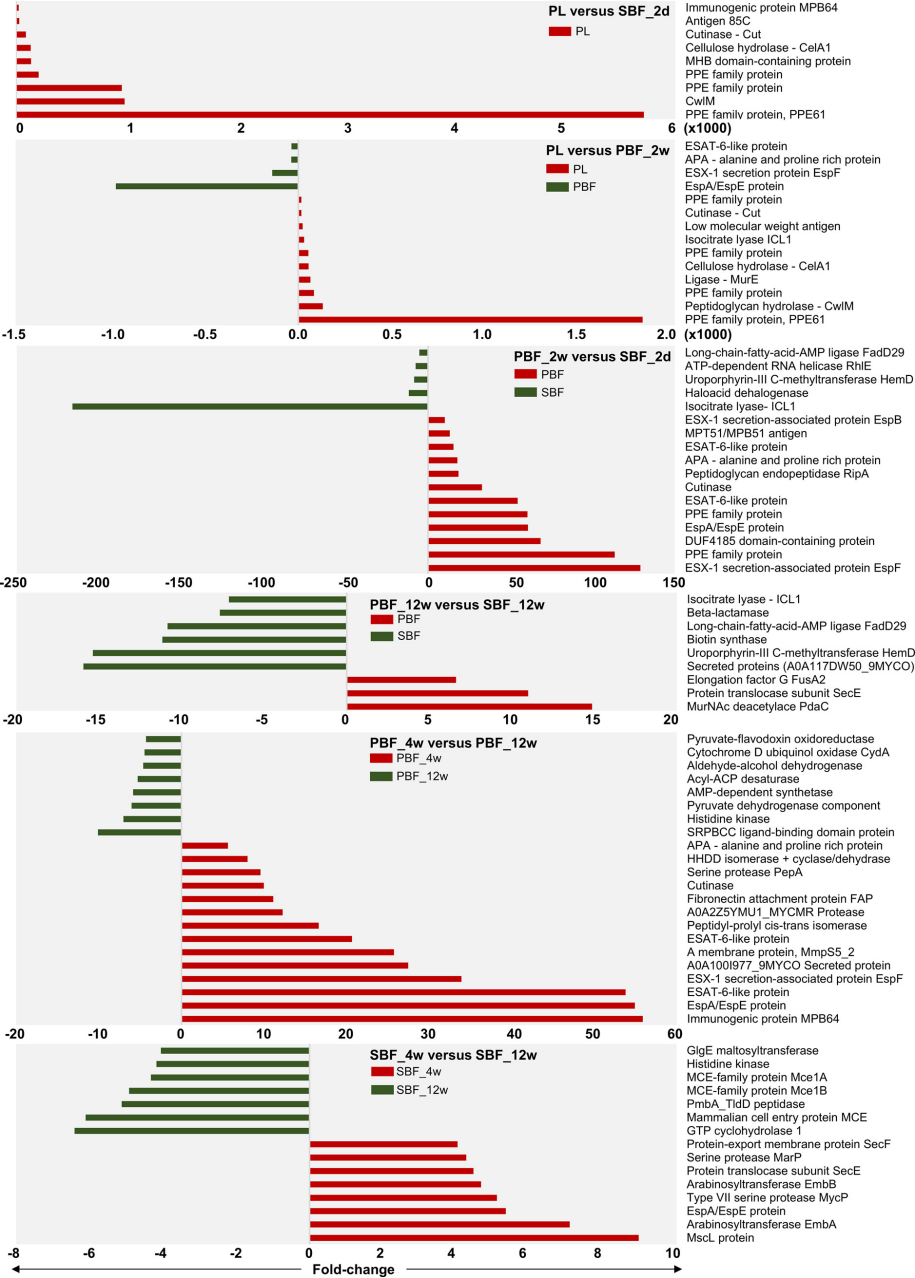
Most significant protein abundance fold changes between the indicated cell types at selected time points. The log_2_-transformed LFQ data were analyzed using Student’s *t* test with permutation-based FDR adjustment. In two top panels, the fold change is times 1,000.

Next, the log_2_ transformed MaxLFQ data were subjected to pairwise comparisons to indicate statistically significant protein abundance changes (see Table S3). Figure 3 shows the greatest growth mode- and time-dependent fold changes related to the PL versus biofilm cells, PBF versus SBF cells, and each biofilm subtype at different time points. Comparison of the PL and both biofilm cells at their first time points of growth (PBF_2w and SBF_2d) indicated the most prominent changes for PPE family proteins (e.g., PPE61) and enzymes involved in cell envelope biogenesis/metabolism (MurE, CwlM, cutinase, and CelA1). Among these, the PPE61 immunogen was ca. 6,000- and 1,800-times more abundant on the PL cells than on the PBF_2w and SBF_2d cells, respectively. CelA1, a β-1,4-cellobiohydrolase known to prevent biofilm growth in Mycobacterium smegmatis and Mtb (11, 18, 19), was detected with 50- and 130-fold higher abundances on the PL cells than on the PBFs at the 1-week time point and the SBFs at the 2-day time point, respectively (Table S3).

10.1128/mSystems.00500-21.6 TABLE S3 Log_2_-transformed MaxLFQ data with minimum of two valid identifications (out of four) in at least one group and statistically significant protein abundance changes between the PL and PBF_2, PL and SBF-2d, PBF_2w_SBF_2d, PBF_12w and SBF_12w, PBF_4w and 12w, and SBF_4w and 12w. Download Table S3, XLSX file, 2.6 MB. Copyright © 2021 Savijoki et al. 2021 Savijoki et al. https://creativecommons.org/licenses/by/4.0/ This content is distributed under the terms of the Creative Commons Attribution 4.0 International license.

Comparison of the PBF_2w and SBF_2d cells indicated Esx1-associated virulence factors (i.e., EspF, EspA/EspE, and ESAT-6) and PPE family immunogens as 30- to 130-fold more abundant from the PBF than from the SBF cells; meanwhile, tricarboxylic acid (TCA)/glyoxylate cycle-associated isocitrate lyase (ICL1) was >200-fold more produced by the SBF than the PBF cells. After 12 weeks, the proteins more abundant in the SBF than in the PBF included an LppP/LprE lipoprotein (ca. 16-fold), HemD involved in the synthesis of vitamin B_12_ (ca. 15-fold), FadD29 contributing to the synthesis of phenolic glycolipids (∼13-fold), β-lactamase able to hydrolase β-lactam antibiotics (ca. 9-fold), and ICL1 catalyzing the glyoxylate shunt-mediated activities (ca. 8-fold). More abundant proteins in the PBF at this time point were identified as a polysaccharide (*N*-acetylmuramic acid [MurNAc]) deacetylase PdaC (ca. 15-fold) and a translocase subunit, SecE (ca. 11-fold).

In the PBF, an MPB64 immunogen, siderophore export accessory protein MmpS5, several Esx1-associated proteins (EspA/EspE and EspF) and adhesins (Ala-Pro-Ala-rich protein APA and fibronectin-binding protein FAP) displayed the most significant abundance decreases at the 12-week time point. In the SBFs, these proteins included a large-conductance mechanosensitive channel protein, Msc, a membrane protein acting as the cells’ safety valve to relieve osmotic pressure, arabinosyltransferases EmbA and EmbB, the Esx1-associated EspA/EspE, and the MycP1 protease. Proteins with the greatest abundance changes after 12 weeks in the SBFs included mammalian entry proteins (MCEs) and an α-1,4-glucan:maltose-1-phosphate maltosyltransferase.

### Decreased CelA1 synthesis is also required for biofilm formation in M. marinum.

**FIG 4:**
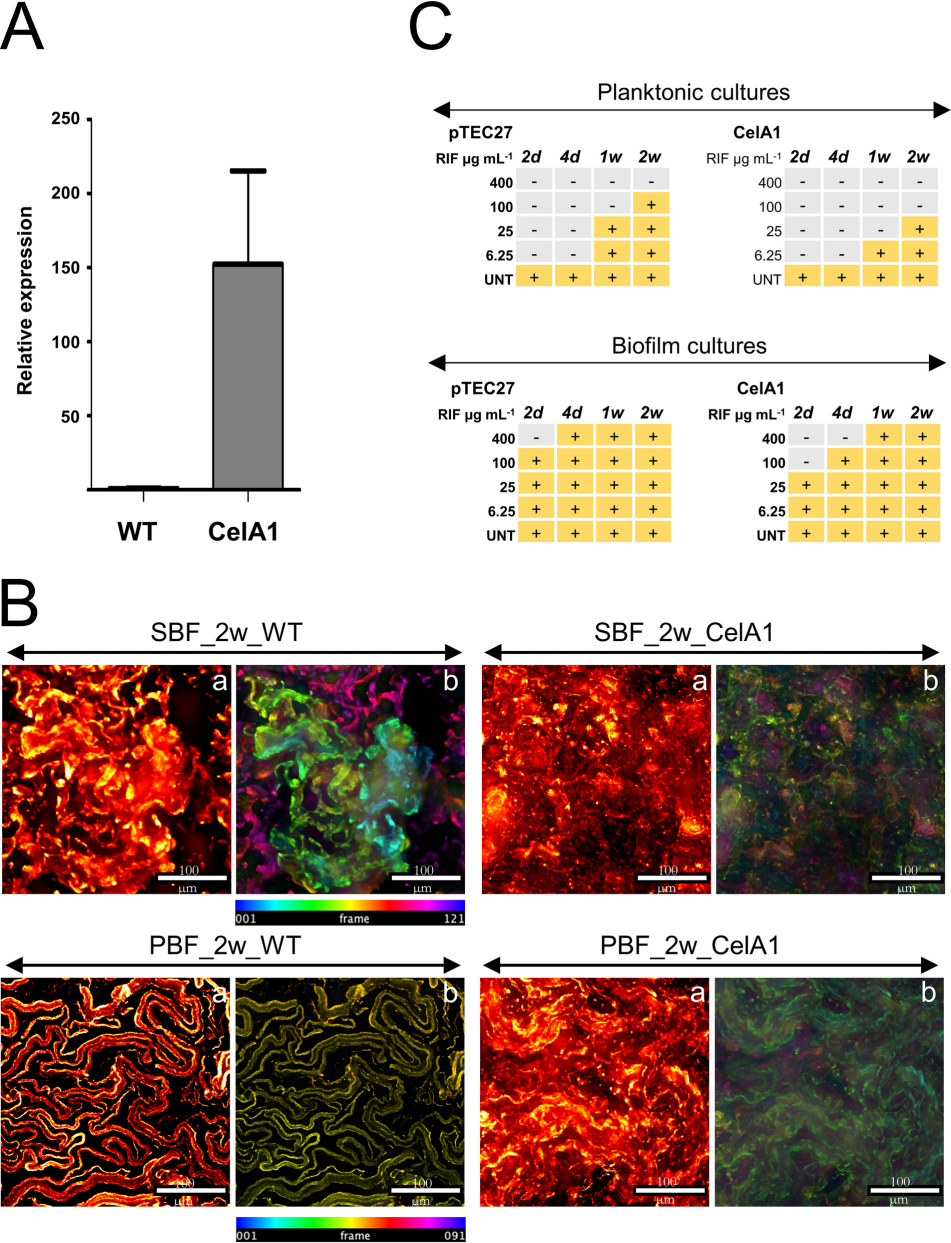
(A) Comparison of relative transcript abundance for *celA1* between the Mmr-CelA1 overexpression strain and the Mmr control strain with pTEC27 (WT). The CelA1 overexpression levels were normalized to the expression level of CelA1 in the WT control. The data were obtained from two technical replicates from two different bacterial clones. The bars represent the standard deviations. CelA1 expression was normalized to the amount of Mmr DNA in each sample. (B) CelA1 overexpression disrupts the biofilm development and the formation of the subtype-specific growth morphologies. The WDeM images are maximum-intensity projections of the 2-week-old Mmr control biofilms (pTEC27, WT) and the Mmr-CelA1 cultures (a), together with color coded by *z* position images (b). Frame interval is 2 μm. (C) The MIC/MBC of rifampicin is reduced in both the PL and biofilms formed with the CelA1 overexpressing strain compared to that in the Mmr control cultures (PTEC27, WT). Rifampicin was added to the liquid cultures 2, 4, 7, and 14 days after the start of the culture. Ten microliters per sample (in triplicates) was plated 7 days after the addition of rifampicin, and CFU were counted 7 days thereafter. One hundred CFU per sample was used as the cutoff limit for bacterial growth. The experiment was carried out three times. The figure shows a representative experiment. −, no growth; +, bacterial growth; UNT, untreated.

As our findings suggest that a lack of CelA could also promote biofilm formation of Mmr, we tested this hypothesis by overexpressing the *celA1* gene in an Mmr strain equipped with pTEC27 with the tdTomato fluorescent marker (29). First, the *celA1* expression level in the transformed Mmr strain was confirmed by quantitative PCR (qPCR), indicating ca. 150-times higher *celA1* transcription than in the control strain carrying an empty pTEC27 (Fig. 4A). Then we analyzed the morphology of both the SBFs and PBFs after 2 weeks using the CelA1 strain with WDeM. As seen in Fig. 4B, the CelA1 strain showed altered morphology compared to that of the Mmr with pTEC27 (wild-type [WT] control strain). After 2 weeks of growth, the CelA1 strain SBF showed a less defined/loss of the lichen-like morphology and lower total thickness than the SBF control with pTEC27. Similarly, CelA1 overproduction in Mmr resulted in disrupted and fuzzy ribbon-like cords associated with PBF-type biofilm growth, as the PBF cells with pTEC27 had well-defined and tight ribbon-like structures.

CelA1 expression was recently linked with biofilm formation, antibiotic tolerance, and virulence in Mtb (9). Therefore, Mmr cells in planktonic and biofilm forms with/without CelA1 overexpression were also exposed to rifampicin to determine the MIC and minimum bactericidal concentration (MBC) for this bactericidal first-line TB drug. Figure 4C shows that in both the planktonic and biofilm cultures, CelA1 overexpression decreased the MIC and MBC, with a clear impact on 2-day-old and 4-day-old biofilms. These results indicate that CelA1 in Mmr impedes biofilm formation and increases the susceptibility of the residing cells to rifampicin.

### Functional pathways specifically induced in planktonic and biofilm cells.

**FIG 5:**
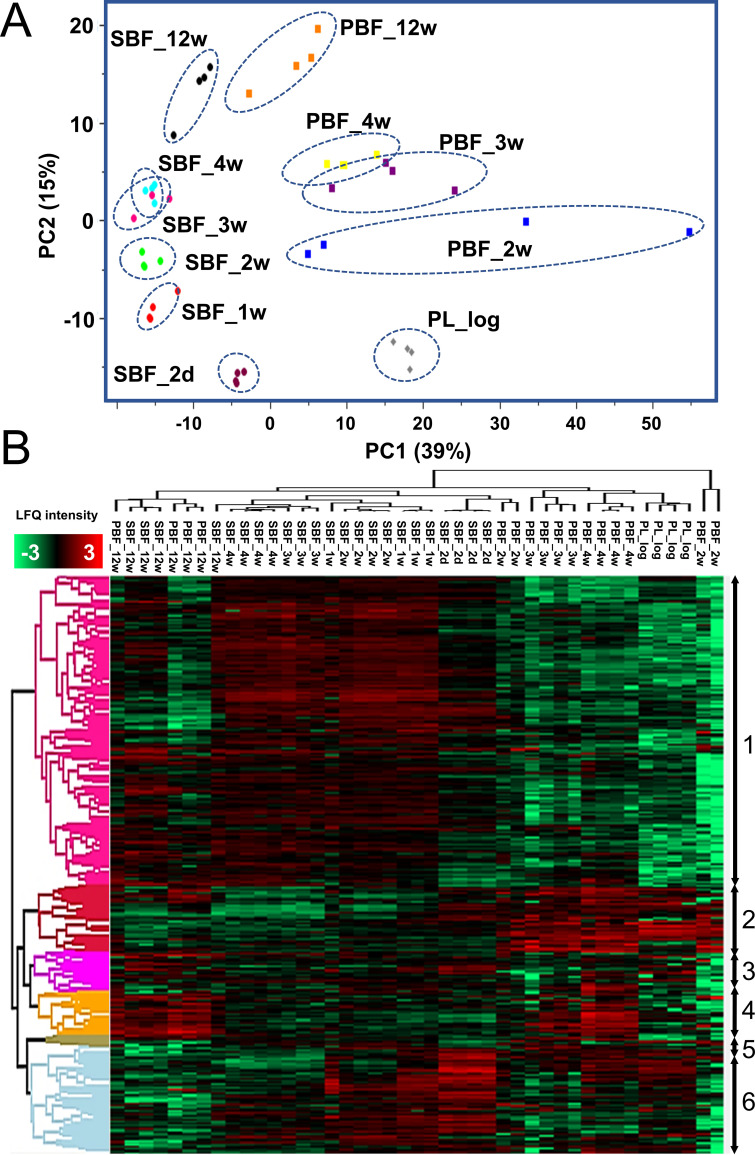
(A) PCA analysis of all detected proteins (based on LFQ intensities excluding one SBF_3w outlier), with PC1 and PC2 indicating growth mode- and time point-dependent changes. (B) Hierarchical clustering of proteins (complete linkage; *n*, 690) with significantly changed expression profiles. Color intensity: red and green indicate higher and lower protein abundances, respectively.

The LFQ proteomic data were next subjected to a principal-component analysis (PCA) for comparing growth mode- and time-dependent protein abundance patterns in the PL cells and aging biofilms. The PCA in Fig. 5A shows clear clustering for each data set except for replicates associated with 2-week-old PBF proteomes, which show greater variation. PC1, separating the samples according to the growth mode, explains 15% of the total variation, while 39% (PC2) of the variation can be explained by the age of the culture. The 2-day-old SBF proteomes formed a clearly distinguishable cluster, while the PL proteomes and proteomes associated with the PBFs between the 2- and 4-week time points showed close clustering. Although the SBF and PBF proteomes differed greatly within the first 4 weeks of growth, these biofilm subtypes appeared to undergo similar proteome changes during the later stages of growth, as proteomes of both subtypes clustered more closely at the 12-week time point. Notably, PBFs during the first weeks (2 to 3 weeks) of growth shared a more similar ECM proteome with that of the PL cells than that of the SBFs under the same conditions.

**FIG 6:**
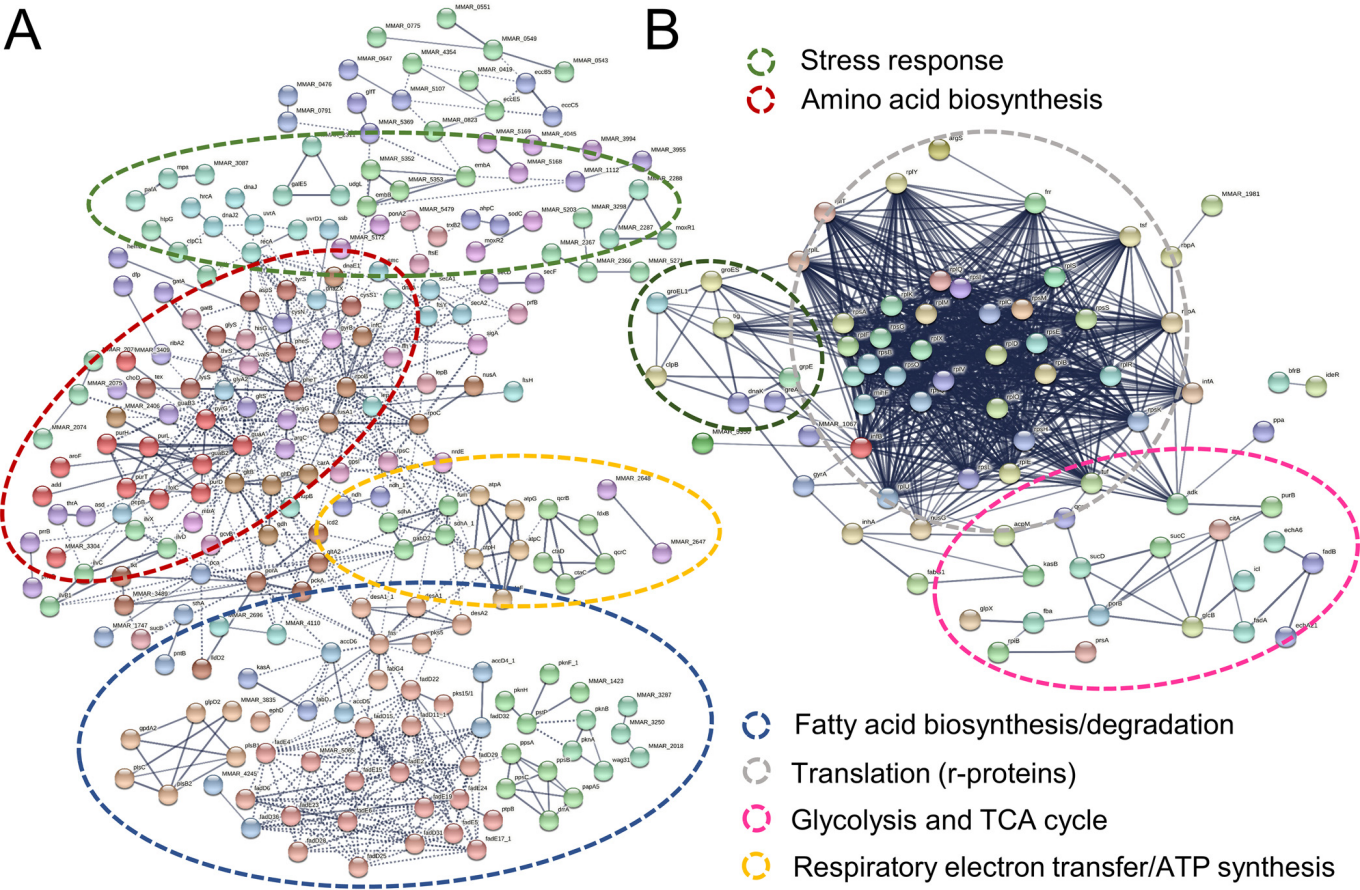
(A) PPI network analysis of cluster 1 proteins (Fig. 5B) with higher abundancies on SBFs between 2 and 4 weeks. Number (no.) of nodes, 368; no. of edges, 3,256; PPI enrichment, *P* < 1.0e−16. (B) PPI network analysis of cluster 6 proteins with higher abundancies in SBFs between 2 days and 2 weeks. Proteins were clustered using MCL with the inflation parameter set to 4.0 (cluster 6) and 6.0 (cluster 1). No. of nodes, 155; no. of edges, 3,024; PPI enrichment, *P* < 1.0e−16. Circles indicate the most enriched protein interactions.

Next, a multisample test (analysis of variance [ANOVA]) was conducted on the normalized LFQ intensity data to investigate growth mode-dependent proteome differences at time points between 2 days and 3 months. A dendrogram/heat map in Fig. 5B shows hierarchically clustered coabundance data for 690 proteins having a statistically significant abundance change in at least one of the conditions tested (see Table S4). Six major clusters were clearly distinguished, among which cluster 1 (*n*, 375) and cluster 6 (*n*, 125) contained the greatest number of proteins, with higher abundances in 1- to 4-week-old SBFs (cluster 1) and 2-day- to 2-week-old SBFs (cluster 6), respectively. STRING (Search Tool for the Retrieval of Interacting Genes/Proteins) enrichment analyses performed on both clusters (see Table S5) indicated the greatest changes for pathways coordinating cell envelope biogenesis/metabolism, energy metabolism, and protein secretion/export. Figure 6A shows a protein-protein interaction (PPI) network for cluster 1 proteins: (i) cytoplasmic proteins with a primary function in amino acid biosynthesis (e.g., Gly, Asp, Tyr, Arg, His, Thr, Ser, Lys, and Phe), purine/pyrimidine metabolism (e.g., PyrG, PurD/L/H, and GuaB), and stress response (HrcA, ClpC/X, DnaJ, HtpG, AhpC, SodC, RecA, and Trx), (ii) proteins involved in cell wall/outer layer and mycomembrane biogenesis/metabolism (e.g., PknA/B, Weg31, CwsA, CwlM, PbpA1a, EmbA/B, KasA, DesA1/2, PpsA/B/D, PcaA, and Fad enzymes), (iii) components of the respiratory electron transport chain (SDH, FMR, and Qcr complex) and ATP synthesis (F_1_F_o_ ATP synthase complex), and (iv) proteins involved in iron storage/homeostasis (ferritin). The PPI network analysis of the cluster 6 proteins indicated the enrichment of metabolic activities related to translation (ribosomal proteins/r proteins), stress response (GroEL/ES, GrpE, DnaK, TF, and ClpB) and the TCA/glyoxylate cycle (e.g., CitA, ICL1, fructose-bisphosphate aldolase [FBA], and GlcB) (Fig. 6B).

10.1128/mSystems.00500-21.7 TABLE S4 Statistically significant protein abundance changes within planktonic cell surfaces and biofilm ECMs. Colored cells in column “Cluster” correspond to those used in the heat map (Fig. 5). Significant changes were calculated using a multiple-sample test (ANOVA model, FDR < 0.05, S0 = 0.1). Color intensity code bar below; blue, low abundance; yellow, high abundance. Download Table S4, XLSX file, 0.9 MB. Copyright © 2021 Savijoki et al. 2021 Savijoki et al. https://creativecommons.org/licenses/by/4.0/ This content is distributed under the terms of the Creative Commons Attribution 4.0 International license.

10.1128/mSystems.00500-21.8 TABLE S5 Functional enrichment analysis (GO, KEGG, InterPro, and Pham) of proteins in clusters 1, 2, and 6 (Fig. 5). Proteins were studied using the STRING database v. 11 with both the rank- and gene set-based approaches (FDR of 0.05). Download Table S5, XLSX file, 0.04 MB. Copyright © 2021 Savijoki et al. 2021 Savijoki et al. https://creativecommons.org/licenses/by/4.0/ This content is distributed under the terms of the Creative Commons Attribution 4.0 International license.

Clusters 2, 4, and 5 (*n*, 144) share coabundance patterns, which indicate increased protein abundances during the first weeks of growth in the PBFs compared to that in the SBFs. These contain virulence-, invasion-, and viability/persistence-related proteins, such as EsxA/B, ESX-EspB/G/M/P/N, Esx5 secretion-associated protease MycP, cutinase (Cut), a lysophospholipase (YtpA), endopeptidase (Lon), heparin binding hemagglutinin (HbhA), and fibronectin binding (Apa), catalase-peroxidase (KatG), and mammalian entry proteins (MCEs). Cytoplasmic proteins were also detected in these clusters (e.g., ICL2, aconitase [ACN], enolase [ENO], glyceraldehyde-3-phosphate dehydrogenase [GAPDH], GPD, Tpi, PGK, malate dehydrogenase [MDH], ClpP1/2, CpsA/D, Trp, Cys, Met, and an 18-kDa β-CA), but their composition differs clearly from those in clusters 1 and 6. In addition, cluster 2 contains virulence-associated ESAT-6-like proteins, TDM-cord factor synthesis-associated Ag85A/C (mycolyltransferases), and an MPT64 immunogen, with higher overall abundancies in the PL and PBF cells than in the SBFs. The remaining cluster 3 (*n*, 47) differs from the other five by proteins with its overall higher abundancies in the PL cells and/or in 4- to 12-week-old PBFs than in the SBFs at the same time points. One of these was identified as a potential trehalase (A0A2Z5YJK7_MYCMR), a glycoside hydrolase that catalyzes the conversion of trehalose to glucose, which had a high abundancy in 4- and 12-week-old PBFs.

**FIG 7:**
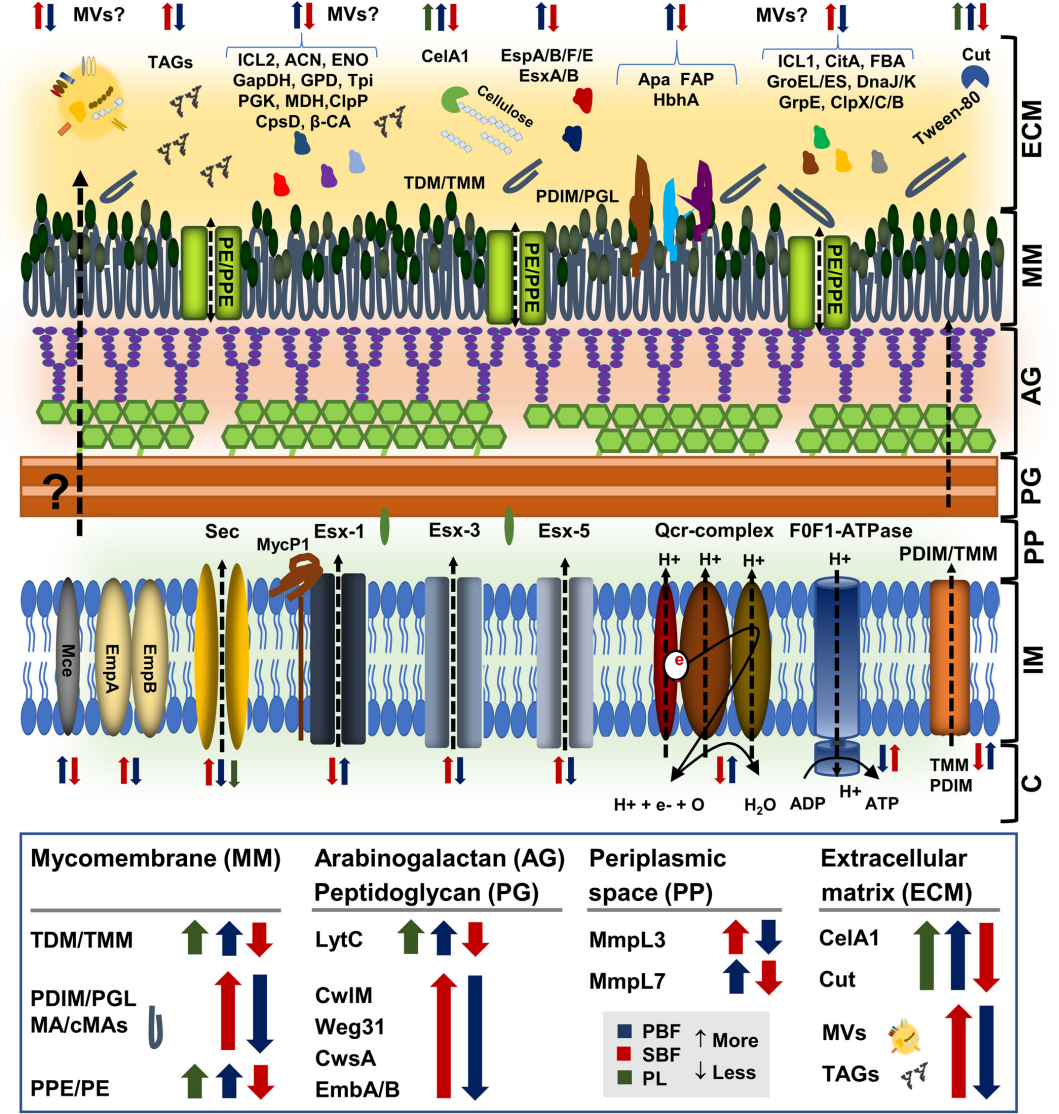
Schematic model of the Mmr cell envelope with key protein abundance changes specific to PL, PBF, and SBF cells. Colored arrows pointing up/down refer to protein abundances/abundance changes within the indicated cell sample types (green, PL; blue, PBFs; red, SBFs). MA, mycolic acids; cMAs, cyclopropanated mycolic acids; TDM/TMM, trehalose-6,6-dimycolate/trehalose monomycolate; PDIM/PGL, phthiocerol dimycocerosates/phenolic glycolipids. C, cytoplasm; IM, inner membrane; PP, periplasmic space; AG, arabinogalactan; PG, peptidoglycan; MM, mycomembrane; ECM, extracellular matrix.

The protein identifications most relevant to biofilm growth and viability identifications are listed in Table S6 according to their predicted functions. The major growth mode-dependent changes associate with the following five functional groups: (i) secretion mechanisms, virulence, and adherence; (ii) cell wall/membrane/lipid biogenesis and metabolism and biofilm formation; (iii) stress response; (iv) TCA/glyoxylate cycles and carbohydrate metabolism; and (v) maintaining redox balance and energy metabolism. An additional schematic model of the mycobacterial cell envelope in Fig. 7 illustrates the key proteome changes relevant to the PL-, SBF-, and PBF-type growth of Mmr.

10.1128/mSystems.00500-21.9 TABLE S6 Key proteome changes within the planktonic cell surfaces and biofilm matrices at different time points of growth. Gradient bar, normalized identification intensity values (average, ≥3). Download Table S6, DOCX file, 0.07 MB. Copyright © 2021 Savijoki et al. 2021 Savijoki et al. https://creativecommons.org/licenses/by/4.0/ This content is distributed under the terms of the Creative Commons Attribution 4.0 International license.

### Time-kill curve analysis for indicating persister cells in maturing biofilms.

As growth mode-dependent differences imply higher persistence/tolerance-associated activities in biofilms than in planktonic cultures, we next validated these findings by exposing both the planktonic and biofilm cells to rifampicin and monitored cell death using a time-kill curve analysis. This method enables the demonstration of an overall slower killing efficacy for tolerant populations or a bimodal time-kill curve that indicates the presence of a persistent bacterial subpopulation (31, 32).

**FIG 8:**
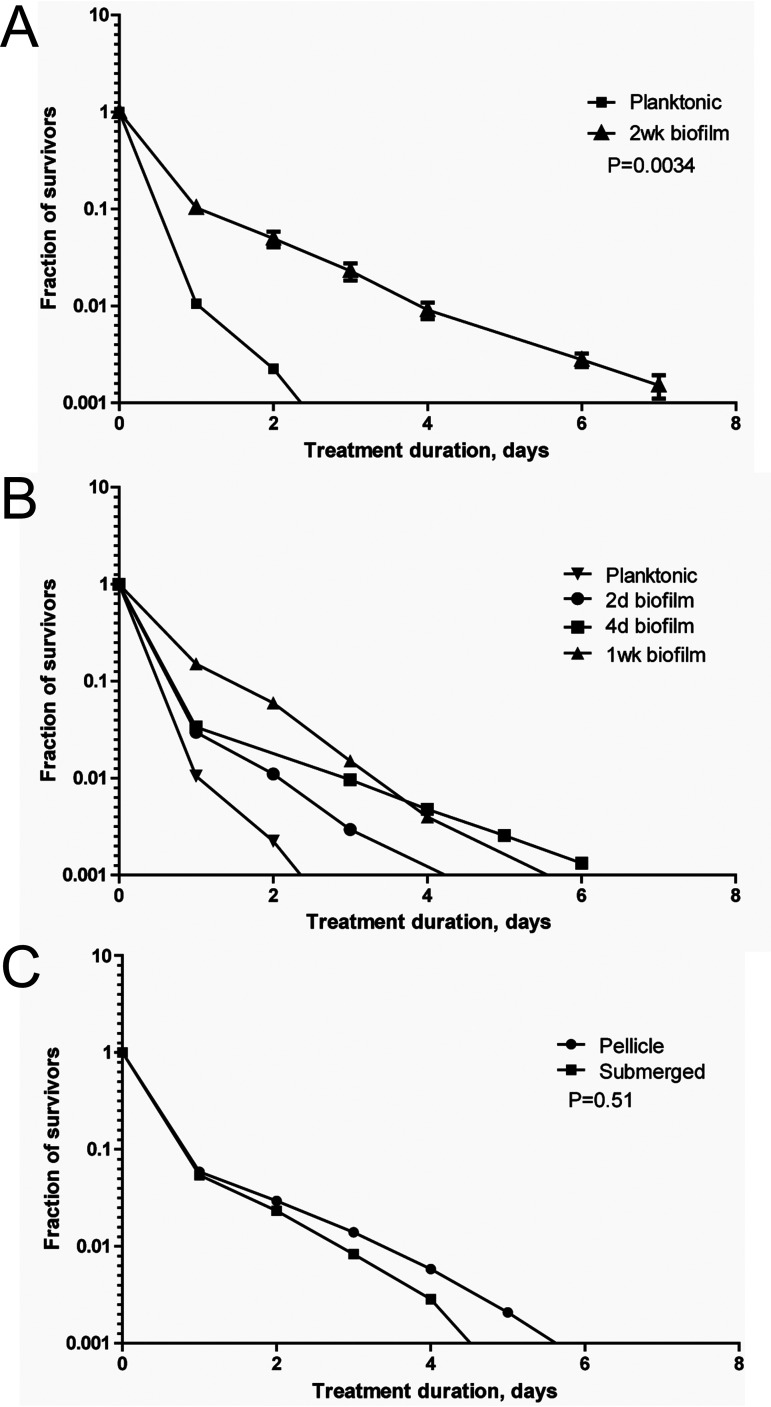
The proportion of persistent bacterial cells increases in Mmr biofilms. Time-kill curve analysis was performed by culturing biofilms from 2 days to 2 weeks and treating the bacteria with 400 μg ml^−1^ rifampicin. The killing kinetics were monitored for 7 days by measuring the bioluminescence signal produced by Lux-Mmr daily. (A) Logarithmic growth phase planktonic cells and 2-week-old biofilm Mmr were treated with 400 μg ml^−1^ of rifampicin. The time-kill curves of the planktonic and biofilm-associated bacteria were significantly different (*P* < 0.0034, log rank test). The means and standard errors of the means (SEMs) from three biological replicates are shown. (B) In biofilms, persistence increases over time and is significantly higher after 1 week than that in planktonic bacteria (*P* = 0.0002, log rank test). Planktonic culture and 2-day-old biofilm show similar killing curves. Means and SEMs from three biological replicates are shown. (C) Two-week-old PBFs and SBFs were tested separately for persistence. The two different biofilm types show no difference in their persistence levels (*P* = 0.51, log rank test). Means and SEMs from three biological replicates are shown.

First, we used a bacterial killing assay with bioluminescence as a readout to quantify the tolerance/persistence in the planktonic cultures and 2-week-old biofilms. The planktonic and biofilm cells were treated with 400 μg ml^−1^ rifampicin (64× MIC), and the rate of bacterial killing was monitored for 7 days. The use of bioluminescence as a readout for killing biofilm-associated bacteria was also assessed using an optical density at 600 nm (OD_600_)-based method (see Fig. S3A). The time-kill curve for the biofilm population was bimodal, showing the faster killing of a susceptible subpopulation followed by slower killing of a persistent subpopulation of cells (Fig. 8A). These results indicate that Mmr biofilms harbor significantly more persister cells than logarithmic phase planktonic populations.

10.1128/mSystems.00500-21.3 FIG S3 (A) The bioluminescence-based readout of the biofilm killing assay was validated using the OD_600_ method to monitor bacterial growth at various time points of growth, showing similar killing kinetics as observed with the bioluminescence measurements of the same samples. Here, the 4-day-old biofilm and 2-day-old planktonic cells were treated with 200 μg ml^−1^ rifampicin. The mean from three biological replicates of the OD_600_-based assay is shown. (B) Mmr growth is accompanied by increased bioluminescence values in maturing biofilms without the antibiotic treatment, even at timepoints of >1 week of biofilm culture. The bioluminescence was measured three times/3 s using EnVision equipment (Perkin Elmer), and the mean of relative light units (RLUs) per second was calculated. Means and SEMs from three biological replicates are shown. Download FIG S3, PDF file, 0.09 MB. Copyright © 2021 Savijoki et al. 2021 Savijoki et al. https://creativecommons.org/licenses/by/4.0/ This content is distributed under the terms of the Creative Commons Attribution 4.0 International license.

Next, the development of persistence in the biofilms was monitored by killing 2-day-, 4-day-, and 1-week-old biofilm cells with 64× MIC rifampicin. Analysis of the time-kill curves showed that persistence increased gradually in the maturing biofilms, reaching a statistically significant increase in 1-week-old biofilms compared to that in the planktonic cells (*P* = 0.0002) (Fig. 8B). In untreated biofilms, the bioluminescence signal level continued to increase well past the 1-week time point, showing that the biofilm-associated mycobacterial population was replicating and/or metabolically active at this stage (Fig. S3B). This indicates that increased persistence is not (mainly) caused by the induction of dormancy or metabolic inactivity. According to our experimental settings, PBFs form later than SBFs and are visually detectable only after 2 weeks. Thus, these data show that a substantial persister subpopulation develops in SBFs by the first week of biofilm development.

To test if the formation of persister cells differs between the two biofilm subtypes, PBFs and SBFs were collected separately and tested with the time-kill assay under 64× MIC rifampicin. After 7 days, the time-kill curves indicated no significant differences in the rates of persistence between the 2-week-old pellicle and submerged biofilms (*P* = 0.51) (Fig. 8C). Thus, our results indicate that the proportion of persisters is greater in >1-week-old Mmr biofilms than in logarithmic planktonic cell populations and that the biofilm-associated persistence increases over time.

## DISCUSSION

### Mmr grows in morphologically distinct biofilm subtypes *in vitro*.

A recent study confirmed that Mtb forms biofilm-like communities *in vivo*, which confers increased tolerance to rifampicin and thus provides an explanation for the chronic nature of TB (11). The present study shows that Mmr grows in two different biofilm subtypes and that reduced CelA1 hydrolase activity is one of the main triggers of biofilm growth and increased tolerance to rifampicin in both biofilm subtypes. Studies on Mtb and M. smegmatis have demonstrated that cellulose filaments are vital structural constituents of mycobacterial biofilm ECMs as well as essential for biofilm formation and the development of tolerance/persistence (9, 11, 18, 19). We also show that the Mmr biofilm subtypes show distinct morphologies, with SBFs containing lichen-like structures and PBFs consisting of ribbon-like cords under the same *in vitro* conditions. Biofilm growth accompanied by cording-like growth morphology is also reported for other mycobacteria and Mtb, in which the surface interactions mediated by, e.g., mycolic acids modulating the mycomembrane/capsule hydrophobicity (11, 33). The proteomic data presented here suggest that subtype-specific changes in cord factor TDM synthesis (mycolyltransferase Ag85), Esx1 secretion, phthiocerol dimycocerosate (PDIM) export (MmpL7), MA cyclopropanation (PcaA/Cma2), and lectin synthesis (33–37) may have affected the mycomembrane composition and thereby contributed to distinct biofilm growth morphologies in Mmr.

### Mmr may use membrane vesicles to deliver proteins in the biofilm ECM.

The LFQ proteomics identified cytoplasmic proteins and proteins associated with the inner membrane/mycomembrane as the largest protein group in both the planktonic and biofilm cells. These findings are supported by studies identifying cytoplasmic proteins in the capsule of another Mmr strain (E11) and by showing that their number increases when mycobacterial cells grow in the biofilms, as demonstrated for Mycobacterium bovis (17, 20). Membrane vesiculation is the most likely explanation for their presence on Mmr cells and within the biofilm ECM, as several reports have demonstrated the presence of membrane vesicles (MVs) on mycobacterial cells (38) as well as trapped in biofilm ECMs in other bacteria (39). In addition, several of the cytoplasmic and inner membrane/mycomembrane proteins detected here, including, e.g., enzymes involved in cell wall synthesis and lipid/fatty acid metabolism, were previously identified in MVs released by Mycobacterium avium 104 in response to starvation (40). Mycobacteria have been shown to form MVs from mycomembrane (mMV) during normal growth (cell lysis/death) and/or from the inner membrane (iMV) by blebbing in response to stress (e.g., iron limitation and anoxia) (38). This report supports the idea that the identified mycomembrane/inner membrane proteins could have also entered the biofilm ECMs by MVs in our study. We further propose that CwlM, an *N*-acetylmuramoyl-l-alanine amidase (41, 42), detected in 1-week-old SBFs, is involved in this process, as weakening the link between the mycomembrane and peptidoglycan has been suggested to stimulate MV blebbing in the mycobacteria (38). Taken together, these findings may explain why more cytoplasmic proteins were detected in this biofilm subtype, as the maturing biofilm cells grow under reduced oxygen tension and anoxia is one of the factors able to trigger membrane vesiculation.

Bacterial MVs are involved in, i.e., cell-cell communication, biofilm formation, virulence, antibiotic resistance, iron scavenging, nutrient acquisition, and modulating the host immune system (43). We detected several cytoplasmic proteins involved in signal transduction (e.g., PknL specific to SBFs and an adenylate cyclase detected only in biofilm ECMs) and enzymes involved in biofilm formation. GroEL1 and fatty-acid synthase system (FAS-I and FAS-II) enzymes were among the detected proteins that coordinate biofilm formation in mycobacteria. The GroEL1 chaperone is involved in the synthesis of mycolic acids (MAs) that eventually become inserted in the mycomembrane as trehalose dimycolates (TDMs) and monomycolates (TMMs) beneath the capsule (14, 21). This chaperone interacts with ketoacyl-acyl carrier protein (ACP) synthase KasA (FAS-II) to modulate the synthesis of short-chain MAs specifically during biofilm formation (21). A lack of GroEL1 has been reported to prevent the biofilm formation and to affect the biosynthesis and composition of MAs in Mycobacterium bovis BCG, whereas the GroEL1 deficiency blocks the formation of mature biofilms of M. smegmatis (21, 24). In addition, the overexpression of KasA and the inactivation of other FAS-II enzymes, such as enoyl-ACP reductase (InhA) and 3-oxoacyl-[acyl-carrier-protein] synthase 2 (KasB), have also been reported to prevent biofilm formation and formation of cords by reducing the cyclopropanation of MAs (14, 21, 25). Here, GroEL1, KasA, and InhA were detected as more abundant in the SBFs, implying that these enzymes could support the initial stages of SBF-type biofilm growth, as GroEL and KasA were detected with the highest abundancies already in the 2-day-old SBFs.

Although no cell lysis was seen during the sample preparation for proteomic analysis (see Table S7 in the supplemental material), we cannot exclude the possibility that some of the cytoplasmic or inner membrane/mycomembrane proteins were released by autolysis during growth. In other Gram-positive bacteria, cytoplasmic proteins reach the extracellular space via regulated autolysis (involving autolysins/peptidoglycan hydrolases), and as soon as the pH of the culture medium drops (due to the active metabolism of the growing cells), many of the released proteins show an enhanced ability to bind to the cell wall and biofilm ECM structures (43–48). SBF cells are exposed to hypoxic conditions, and oxygen limitation acidifies the biofilm matrix (48), allowing for a more efficient interaction between the cytoplasmic proteins and biofilm ECM structures. Thus, this could explain the presence of r proteins as the largest cytoplasmic protein group already in 2-day-old SBFs; the strong positive charge of these proteins has been proposed to mediate electrostatic interactions with anionic cell surface components, which promotes cell aggregation and biofilm stabilization (48). Since the exposed mycomembranes with MAs as the major components create a condition stimulating an interaction with many cytoplasmic proteins, pH-dependent binding with the cell surface components could also explain why cytoplasmic proteins were detected in Mmr cells grown on Tween 80.

10.1128/mSystems.00500-21.10 TABLE S7 Colony ability of nonshaved and enzymatically shaved biofilm and planktonic cells. Biofilm cells were cultured for 2 weeks and planktonic cells for 2 days, as described in Materials and Methods. Cells in three biological replicates were suspended in trypsin/Lys-C digestion buffer and then divided into two aliquots; the first aliquot was taken as the nonshaved cell control containing only the digestion buffer, and the second aliquot of cells was treated with the trypsin/Lys-C enzyme. After 20 min of incubation at 37°C, the cell suspensions were suspended gently in phosphate-buffered saline PBS containing 0.2% Tween 80 (vol/vol), and serially diluted cells were spotted as four technical replicates (10 μl each) on an agar plate. After 1 week of cultivation at 37°C, the colonies were calculated and compared under different conditions. nd, colonies could not be counted due to the presence of cell aggregates. Download Table S7, XLSX file, 0.02 MB. Copyright © 2021 Savijoki et al. 2021 Savijoki et al. https://creativecommons.org/licenses/by/4.0/ This content is distributed under the terms of the Creative Commons Attribution 4.0 International license.

### Biofilm subtypes differ in terms of secreted virulence and adhesion factors.

The proteomics data indicated that the mycomembrane-associated PPE/PE family proteins were remarkably greater in number in the PL cells than in the PBFs or SBFs, indicating that Mmr in a single-cell state could more readily interact with the host and modulate the host immune response and/or nutrient transport (49, 50). PL cells were cultured in the presence of Tween 80, which, in detaching the mycobacterial capsule (17), most likely helped identify these immunogens. Tween 80 can also induce alterations in the morphology, pathogenicity, and virulence of mycobacteria (51). For example, genes encoding lipases and cutinases have been shown to be significantly upregulated in Mtb in response to this nonionic surfactant. Our data are in line with this by showing that several lipases/cutinases, with a likely ability to hydrolyze Tween 80, were more abundant in PL cells than in biofilms. As Tween 80 is considered to mimic a lipid-rich milieu of macrophages (51), the detected PL proteome changes here may reflect a metabolic adaption to conditions faced *in vivo*.

Our findings also suggest that Mmr uses different T7SS pathways in SBFs and PBFs for virulence and adherence. For example, the Esx1 secretion components and substrates (EsxA/B, EspB, EspF, EccA1, EspG1, EspH, EspL, and MycP) were detected as more abundant in the PBFs, while those associated with Esx5-type secretion were overall more abundant in the SBFs (Ecc, EspG, and PPE/PE proteins). Both secretion pathways can contribute to virulence and subverting the host immune system in Mtb (52). The major subtype-dependent differences between the PBFs and SBFs were related to invasion and adherence, including the MCE proteins, fibronectin-binding APA, and HphA, which can modulate host cell signaling as well as aid adhesion or entry into host cells (53–55). All these proteins were significantly more produced by the PBFs than the SBFs, and, in the case of MCEs, may also involve MVs, as these adhesins are located on the inner membrane of the mycobacterial cell wall. HphA also has implications in promoting cell-cell aggregation in Mtb (56), suggesting that this adhesin could also contribute to cording during PBF-type growth.

### Biofilm subtypes use different tolerance- and persistence-conferring mechanisms.

Tolerance is defined as the extent of time that bacteria can survive in the presence of a high antibiotic concentration (31), whereas persisters are a subpopulation of phenotypically drug-tolerant cells that do not grow in the presence of an antibiotic (32). We show that antibiotic killing of biofilm cells occurs at a significantly lower rate than for PL cells. The time-kill curve indicated the temporally increased formation of a persistent subpopulation with slower killing kinetics as well as the formation of persisters in SBFs already after 1 week. At this stage, Mmr biofilms remained metabolically active and replicating, indicating that persistence develops due to phenotypic differentiation during biofilm growth rather than via the induction of dormancy.

The proteomic findings suggest that Mmr could use both overlapping and subtype-specific mechanisms for increasing its tolerance and persistence, in which MVs or other nonclassical routes for protein export may play a role. Here, most significant proteome changes related to cytoplasmic and inner membrane/mycomembrane proteins and included enzymes/proteins involved in the TCA cycle and glyoxylate shunt, mycolic acid synthesis stress response, and energy and redox metabolisms. A recent transcriptome analysis of another nontuberculous mycobacterial model, Mycobacterium abscessus, supports our findings; biofilm growth activated the glyoxylate shunt, redox metabolism, and the MA synthesis-associated elongation and desaturation pathways. The TCA cycle-associated enzyme CitA was recently reported to control the asymmetric cell division in Caulobacter crescentus (57). This process has also been shown in mycobacteria to lead to the formation of heterogenous cell populations in biofilms, macrophages, and granulomatous lesions (7, 58, 59). Here, our findings indicated the presence of this enzyme in 1-week-old SBFs, suggesting that asymmetric cell division occurs before the PBFs are formed. Moreover, arabinosyltransferases EmbA and EmbB, involved in the polymerization of arabinogalactan, were also detected with high abundances in SBFs by 1 week onward, suggesting that strengthening the arabinogalactan could further help residing cells, including the persisters, increase their tolerance to rifampicin, as demonstrated with Mtb persisters under hypoxia (60). Taken together, these findings strengthen the hypothesis that persisters are indeed formed in 1-week-old SBFs and support the results obtained with the biofilm killing assay in SBFs at this time point.

We also suggest that cells in PBFs use different TCA cycle enzymes, such as aconitase (ACN), malate dehydrogenase (MDH), enolase (ENO), and/or fructose-bisphosphate aldolase (FBA), to maintain long-term survival. In other Gram-positive bacteria, these enzymes belong to known moonlighting proteins with established secondary roles outside the bacterial cell (e.g., adhesion) (30). In mycobacteria, these enzymes have been reported to contribute to increased viability or persistence (61–63). The associated glyoxylate shunt could also be involved (64), as isocitrate lyase 1 (ICL1) was detected as more abundant in the SBFs, implying that this enzyme could help residing cells increase their antioxidant defense and antibiotic tolerance (65). In contrast, ICL2 was produced more in the PBFs, which may help the cells to survive under starvation conditions when fatty acids are used as the primary carbon source (66). This is in line with the temporally increased production of diacylglycerol *O*-acyltransferase (Tgs1) in PBFs, which can promote the accumulation of triacylglycerols (TAGs), a process that has been considered a hallmark feature of persisting Mtb/latent TB and a long-term energy source for Mtb and has been found in substantial amounts in the mycobacterial cell wall (67, 68). The detection of trehalase as significantly more abundant in 4- to 12-week-old PBFs strengthens the idea that cells within this biofilm subtype suffer from nutrient stress and activate trehalose salvage/recycling to promote redox and energy homeostasis, as seen under carbon limitations in Mtb (69). These findings may also explain the detection of proteases, chaperones, and assisting stress proteins in high numbers in the biofilm ECMs, including, e.g., the proteases Clp/Lon and the cold shock protein CpsD, with known implications in stringent response, persistence, and/or postantibiotic recovery (70–72). These proteins were detected here as more abundant in the PBFs than in the SBFs, implying that these pathways are preferred in PBFs to maintain viability.

A recent study comparing high numbers of persister Mtb mutants using genomics and transcriptomics indicated a significant upregulation of energy production pathways and pathways involved in redox reactions (oxidoreductase) (73). The ECM proteome changes occurring during SBF-type growth are in line with this report, as the components of the respiratory electron transfer chain (cytochrome *bc*_1_ complex, cytochrome *c* terminal oxidase, and F_o_F_1_ ATPase synthase) were detected as more abundant in the SBFs facing more hypoxic conditions than in the PBFs. Our findings also agree with previous reports showing that the electron transfer chain is essential for maintaining ATP homeostasis and the viability of nonreplicating/persistent Mtb cells under hypoxia (74–76). In addition, we show that both redox and iron metabolism could also play a biofilm subtype-specific role in helping the cells cope with hypoxia/aeration-related stress (77); several oxidoreductases, thioredoxin, and a superoxide dismutase (SOD) were overall more abundant in the SBFs, and a catalase-peroxidase (KatG) and alkyl hydroxyperoxidases (AhpCF) were more abundant in the PBFs. These enzymes have been shown to protect Mtb against oxidative stress by the reduction of superoxide radicals into less toxic intermediates for inhibiting autophagy, apoptosis, and cellular damage (78). Iron-storing proteins ferritin (BfrB) and bacterioferritin (BfrA) can confer increased redox resistance to Mtb and protect the cells against oxidative stress and hypoxia, respectively (79). Here, these iron-storing proteins displayed biofilm subtype-specific abundance changes, implying that SBFs could rely on BfrB to cope with hypoxia and PBFs could rely on BrfA to help cells grow at the air-liquid interface.

### Conclusions.

The present study reports an in-depth view of ECM proteome changes occurring in Mmr ATCC 927 during biofilm growth *in vitro* from 2 days to 3 months. We show that this nontuberculous mycobacterial model forms SBFs already after 2 days, whereas the formation of detectable PBFs was observed after 2 weeks of growth in the absence of Tween 80. Both biofilm subtypes were formed physically under the same conditions with clearly distinct growth morphologies: SBFs with lichen-like structures and PBFs with ribbon-like cords. We show that reduced CelA1-mediated cellulose hydrolysis is necessary to establish proper biofilm growth, growth morphology, and increased tolerance to rifampicin for both biofilm subtypes. The formation of persisters in both biofilm subtypes and increased tolerance were further confirmed by the newly established bioluminescence-based time-kill assay, which provides an effective tool for quantifying tolerance and persistence of Mmr. The proteomic findings imply that subtype-dependent changes in MA synthesis and modification, Esx1-type secretion, and the production of specific adhesins were the major drivers of distinct biofilm growth morphologies. We also propose that pathways associated with MA biosynthesis, development of tolerance/persistence, and oxidative/redox stress are differentially used in PBFs and SBFs to maintain prolonged viability. Possible explanations for these differences include the different oxygen tensions encountered by the biofilm subtypes, differences in membrane vesiculation activities, and/or other nonclassical pathways for protein export. Taken together, this is the first study reporting on ECM proteome dynamics in maturing mycobacterial biofilms and predicting biofilm subtype-specific changes in cell-cell communication, biofilm matrix formation, virulence, and tolerance/persistence. This is also the first time that the kinetics of persistence have been explicitly measured from mycobacterial biofilms.

## MATERIALS AND METHODS

### Preparing bacterial cells for surface proteomics.

Mycobacterium marinum (ATCC 927) with the pTEC27 plasmid expressing the red fluorescent protein tdTomato (Addgene number 30182, http://n2t.net/addgene:30182) (29) was precultured on Middlebrook 7H10 plates with 10% (vol/vol) oleic albumin dextrose catalase (OADC) enrichment (Fisher Scientific, NH, USA) and 0.5% (vol/vol) glycerol at 29°C for 1 week. For planktonic cultures, an inoculum of Mmr was transferred into a Middlebrook 7H9 medium supplemented with 10% (vol/vol) ADC (Fisher Scientific, NH, USA), 0.2% (vol/vol) glycerol, and 0.2% (vol/vol) Tween 80 (Sigma-Aldrich, MO, USA), and the cells were cultured at 29°C in cell culture flasks with filter caps. After 3 days of incubation, the cell cultures were diluted to obtain an OD_600_ of 0.042, and the dilutions were cultured for an additional 2 days at 29°C until harvesting. For the biofilm cultures, a Middlebrook 7H9 medium with the ADC growth supplement but without Tween 80 or glycerol was used. The inoculum was cultured for 3 days at 29°C until the OD_600_ reached 0.45. The cell cultures were diluted 1:40, and the dilutions were divided into 10-ml aliquots. The cap of each tube was sealed with Parafilm M laboratory wrapping film, and the cultures were incubated at 29°C. Planktonic and biofilm cell samples (SBFs and PBFs separately) were collected at the time points indicated in Fig. 2A. All the cultures were performed in quadruplicates. Planktonic cells were harvested by centrifugation (3 min, 5,000 × *g*, 4°C), and the pelleted cells were suspended gently in ice-cold buffer (100 mM bis-Tris, pH 6.5) to remove interfering/nonspecifically bound proteins. This step prevents the detachment/removal of cytoplasmic moonlighters bound to the cell surfaces/biofilm ECM (43–46, 79, 80). The PBFs were collected with an inoculation loop, the extra medium was removed by pipetting to avoid cross-biofilm-type contamination, and the SBFs were harvested by pipetting/scraping. The PBFs and SBFs were collected in separate Eppendorf tubes in ice-cold buffer (100 mM bis-Tris, pH 6.5). Cells (planktonic and biofilm cultures) were pelleted by centrifugation (3 min, 5,000 × *g*, 4°C), and the washed cells were suspended gently in 95 μl of 100 mM triethylammonium bicarbonate (TEAB; pH 8.5) for the enzymatic shaving reaction.

### Trypsin/Lys-C shaving of planktonic and biofilm cells.

Peptides from cell surface/biofilm ECM-associated proteins were released via a trypsin/Lys-C mix (Promega) at a final concentration of 50 ng μl^−1^, and the digestions were incubated at 37°C for 20 min. The method was validated by counting the number of colonies formed by the planktonic/single and biofilm cells treated with/without the enzyme mix (see Table S7 in the supplemental material). The released peptides and the enzymes were recovered by filtration through a 0.2-μm acetate membrane (Costar Spin-X centrifuge tube filter; Corning Inc., Corning, NY, USA) by centrifugation (8,000 × *g*, 3 min, 20°C). Flowthroughs were incubated for 16 h at 37°C. The concentration of released peptides in each sample was measured with a NanoDrop 2000 spectrophotometer (Thermo Scientific). Digestions were terminated with 0.6% (vol/vol) trifluoroacetic acid (TFA) (Sigma-Aldrich), and the peptides were purified using ZipTip C_18_ (Millipore) according to the manufacturer’s instructions and dried using a miVac centrifugal vacuum concentrator (Genevac).

### LC-MS/MS analysis.

The peptides were dissolved in 0.1% (vol/vol) formic acid (FA) and analyzed with nanoscale LC-MS/MS using an Easy-nLC 1000 nano-LC system (Thermo Scientific) coupled with a quadrupole Orbitrap mass spectrometer (Q Exactive; ThermoElectron, Bremen, Germany) as previously reported (80). The obtained MS raw data were processed via MaxQuant software (version v.1.6.1.0) with the built-in search engine Andromeda (81, 82), using a protein database comprising all 5,564 Mmr protein sequences (UniProt proteome up000257451, genome accession PEDF01000000), both forward and reverse. Carbamidomethyl (C) was set as fixed, and methionine oxidation was set as a variable modification. Tolerance was set to 20 ppm in the first search and 4.5 ppm in the main search. Trypsin without the proline restriction enzyme option and with two allowed miscleavages was used. The minimal unique plus+ razor peptide number was set to 1, the false-discovery rate (FDR) was set to 0.01 (1%) for peptide and protein identification, and LFQ with default settings was used. The mass spectrometry proteomics data were deposited in the ProteomeXchange Consortium via the PRIDE (83) partner repository with the data set identifier PXD02010.

### Proteome statistics and bioinformatics.

The identified Mmr proteins were manually curated by characterizing hypothetical and tentatively annotated proteins with the aid of the Basic Local Alignment Search Tool (BLAST) program from the National Center for Biotechnology Information (NCBI) (84–86) combined with CDD/SPARCLE conserved domain identification (87) and SmartBLAST (UniProt) searches. General protein functions were annotated using the Gene Ontology (GO) database (88). Isoelectric points (pIs) and molecular weights (MWs) for the identified proteins were predicted using EMBOSS Pepstats (89) at https://www.ebi.ac.uk/Tools/seqstats/emboss_pepstats/. The presence of possible protein secretion motifs (classical and nonclassical) for all the predicted and identified proteins was obtained with SignalP4.1 (90) (http://www.cbs.dtu.dk/services/SignalP/) and SecretomeP 2.0/SecP (91) (http://www.cbs.dtu.dk/services/SecretomeP/). The presence of transmembrane spanning domains/helices (TMDs) was determined with the TMHMM Server v. 2.0 at http://www.cbs.dtu.dk/services/TMHMM/ (92, 93) for the identified proteins.

For indicating statistically significant abundance changes, the log_2_-transformed LFQ data were analyzed in Perseus v.1.6.2.3 (94) using a Student’s *t* test with permutation-based FDR adjustment. For the multivariate analyses, the missing values were replaced by imputed values from the normal distribution (width, 0.3; down shift, 1.8) and then normalized (Z-score) prior to ANOVA for multisample testing (S0 set to 0.1 and a permutation-based FDR of 5%) and hierarchical clustering/PCA. STRING protein interaction network and functional enrichment analyses (GO, KEGG, InterPro, and Pham) were studied using the STRING database v. 11 (95). Interaction scores were set to high (0.700) confidence, and the interacting proteins were clustered using Markov clustering (MCL) with the inflation parameter set to 4.0 to 6.0. Functional enrichments were statistically assessed with both rank- and gene set-based approaches (FDR of 0.05).

### Creation of the CelA1 overexpression construct in Mmr.

The Mmr CelA1 overexpression strain was created by ordering the MMAR_0107 open reading frame in the pUC57 vector with appropriate restriction sites from GenScript and subcloning the construct into the pTEC27 vector (Addgene) (29), which carries the red fluorescent protein tdTomato. The sequence of the plasmid was confirmed by sequencing. The resulting plasmid was transformed into an electrocompetent Mmr ATCC 927 strain. Transformants were selected on Middlebrook 7H10 agar plates containing 10% (vol/vol) OADC enrichment, 0.5% (vol/vol) glycerol, and 75 μg ml^−1^ hygromycin.

### RNA and DNA extractions.

For RNA and DNA extractions, the *celA1* overexpression strain and Mmr were precultured on Middlebrook 7H10 plates and transferred into the Middlebrook 7H9 medium described above (75 μg ml^−1^ hygromycin for the CelA1 strain). After 3 days, the bacterial cells were harvested, pelleted, and homogenized in TRI reagent (Thermo Fisher Scientific, NH, USA) with ceramic beads using a PowerLyzer24 (MO BIO, CA, USA). After homogenization, the samples were sonicated for 9 min, and the RNA and DNA were extracted according to the manufacturer’s instructions.

### *celA1* expression and the quantification of mycobacterial loads by qPCR.

Prior to qPCR analysis, RNA was reverse transcribed into cDNA with a reverse transcription kit (Fluidigm, CA, USA) according to the manufacturer’s instructions. *celA1* expression was measured using soFast EvaGreen supermix with the low ROX qPCR kit (Bio-Rad, CA, USA) and the CFX96 qPCR system (Bio-Rad). The primers used for *celA1* were (forward) 5′-ACACTCCGCAGTCCTACT-3′ and (reverse) 5′-TAGAGCGTCAGAATCGGC-3′. The number of mycobacterial cells in the sample was quantified using the SensiFAST SYBR no-ROX qPCR kit (Bioline, London, UK) on bacterial DNA according to the manufacturer’s instructions. The primers used for Mmr quantification were targeted against the 16S-23S locus AB548718 (forward, 5′-CACCACGAGAAACACTCCAA-3′; reverse, 5′-CACCACGAGAAACACTCCAA-3′). Each bacterial quantification qPCR run included a standard curve of the known amounts of Mmr DNA. The mycobacterial cell number in each sample was used to normalize *celA1* expression.

### Widefield deconvolution microscopy of Mmr biofilms.

PBFs and SBFs formed by Mmr with pTEC27 (WT), expressing the red fluorescent protein tdTomato (29), and Mmr overexpressing CelA1 were prepared as follows. Briefly, the cells were incubated at 29°C, and the surface-attached cells were imaged at 7, 14, and 21 days after dilution. *In situ* imaging of the SBFs was conducted with Nikon FN1 upright epifluorescence microscope equipped with a 20×/0.8 dry lens objective, Hamamatsu ORCA-Flash4.0 V3 digital complementary metal-oxide-semiconductor (CMOS) camera, and CoolLED pE-4000 light source. tdTomato was excited with a 550-nm light-emitting diose (LED), and fluorescence was collected with a 617/73 band-pass emission filter. Image stacks were collected with 2-μm intervals (*x*-*y* pixel size, 325 nm). The data were deconvolved with Huygens Essential deconvolution software (SVI, Amsterdam, Netherlands) using a 200-iteration limit, signal-to-noise ratio of 30, and quality threshold of 0.01.

### Biofilm tolerance assays.

The role of CelA1 overexpression in the antibiotic tolerance of Mmr was assessed as follows. First, the *celA1* overexpression strain and pTEC27 control strain were cultured for 1 week on 7H10 plates (10% OADC and 0.5% glycerol plus 75 μg ml^−1^ hygromycin) and then transferred in a Middlebrook 7H9 medium supplemented with 10% ADC and 75 μg ml^−1^ hygromycin) at an OD_600_ of 0.1 to initiate biofilm growth. Aliquots of bacterial suspension (192 μl per well) were added to 96-well-plates in triplicates, sealed with parafilm, and incubated at 29°C in the dark. Planktonic cultures grown in the presence of 0.2% (vol/vol) Tween 80 were used as controls. Eight microliters of antibiotics per well was added 2, 4, 7, and 14 days after the start of the liquid culture. The final antibiotic concentrations used were 400, 100, 25, and 6.25 μg ml^−1^ for the rifampicin TOKU-E solution. Untreated wells were used as controls. Ten microliters per sample was plated on 7H9 plates (10% OADC, 75 μg ml^−1^ hygromycin) 1 week after the addition of antibiotics. The plates were incubated at 29°C for 7 to 9 days, and the colonies were counted.

### Biofilm persistence assays.

Mmr (ATCC 927) with a bioluminescence cassette in the pMV306hsp+LuxG13 plasmid was used for antibiotic tolerance assays. pMV306hsp+LuxG13 was provided by Brian Robertson and Siouxsie Wiles (Addgene plasmid number 26161; http://n2t.net/addgene:26161). To measure the kinetics of bacterial killing, the bioluminescent Mmr strain was first cultured on Middlebrook 7H10 agar (Sigma-Aldrich) supplemented with 0.5% (vol/vol) glycerol (Sigma-Aldrich) and 10% (vol/vol) OADC enrichment (Becton, Dickinson) at 29°C for 7 days in the dark. To initiate biofilm formation, the Mmr cells were suspended in Middlebrook 7H9 broth (Sigma-Aldrich) supplemented with 10% (vol/vol) ADC enrichment (Becton, Dickinson) at a starting OD_600_ of 0.1. Planktonic cultures were prepared in the same way except that the medium contained 0.2% (vol/vol) glycerol (Sigma-Aldrich) and 0.2% (vol/vol) Tween 80 (Sigma-Aldrich). Bacterial suspensions (192 μl per well in triplicates) were divided into white 96-well plates (Perkin Elmer). The biofilm cultures were sealed with laboratory film and incubated at 29°C in the dark to the desired ages. Rifampicin solution (TOKU-E) in water at a final concentration of 400 μg ml^−1^ corresponding to 64× MIC was added to the bacterial suspensions and incubated for 7 days at 29°C in the dark. The bioluminescence signal was measured with an EnVision plate reader (Perkin Elmer) as a readout for bacterial survival three times for 3 s per well daily from a white 96-well plate for 7 days. The background signal from medium-only wells was first subtracted from the sample wells, and an average of the three measurements normalized with the starting bioluminescence signal was used to draw time-kill curves of the bacterial population in the biofilms at different maturation stages.

To compare the level of persistence/tolerance in the PBFs and SBFs, Mmr was cultured in a total volume of 10 ml at the starting OD_600_ value of 0.1. After 2 weeks, the biofilms were collected separately from the tubes by lifting the pellicle with a 1-μl inoculation loop coupled with careful pipetting. The pellicle and submerged biofilms were centrifuged at 10,000 × *g* for 3 min, the supernatants were collected, and the wet weight of the bacterial mass was measured. The bacterial cells were suspended into previously collected spent medium at the concentration of 15 mg ml^−1^, vortexed briefly, and divided into white 96-well plates (Perkin Elmer) with 192 μl of cell suspension per well in triplicates. Eight microliters of TOKU-E solution at a final concentration of 400 μg ml^−1^ was pipetted into the bacterial suspension. Liquid cultures were incubated for 7 days at 29°C in the dark, and the bioluminescence signal was measured daily with an EnVision plate reader (Perkin Elmer) three times for 3 s per well. The background signal from the medium-only wells was first subtracted from the sample wells, and an average of the three measurements was normalized with the starting bioluminescence signal measured just before adding the rifampicin. The statistical significance of the differences between the time-kill curves was tested with a log rank test using Prism5 software (GraphPad).
