# Supplementary figures and images for: Surface-Shaving Proteomics of Mycobacterium marinum Identifies Biofilm Subtype-Specific Changes Affecting Virulence, Tolerance, and Persistence
Source: mSystems. 2021 Jun 22;6(3):e00500-21. doi: 10.1128/mSystems.00500-21 (PMC8269238; doi:10.1128/mSystems.00500-21)

# A

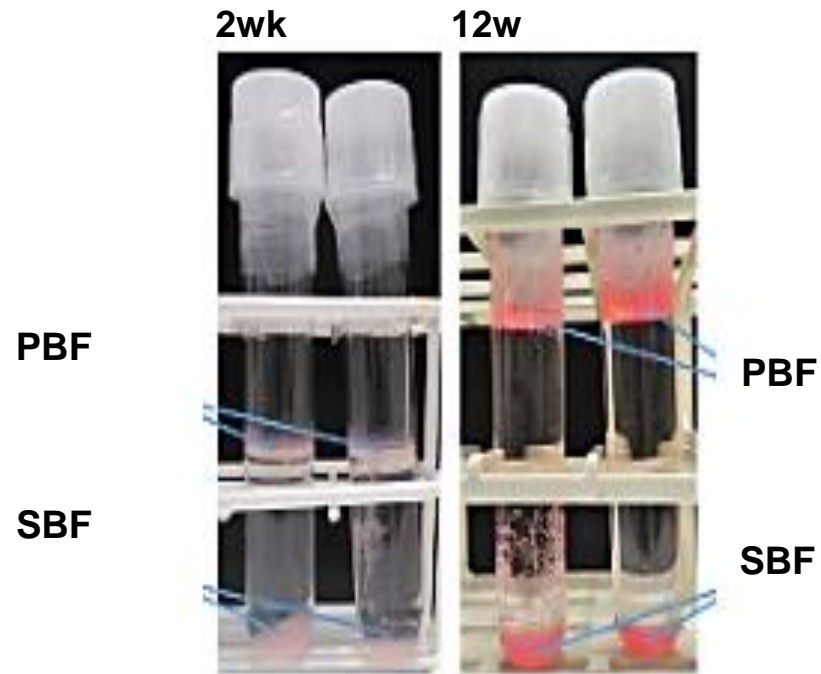

# B

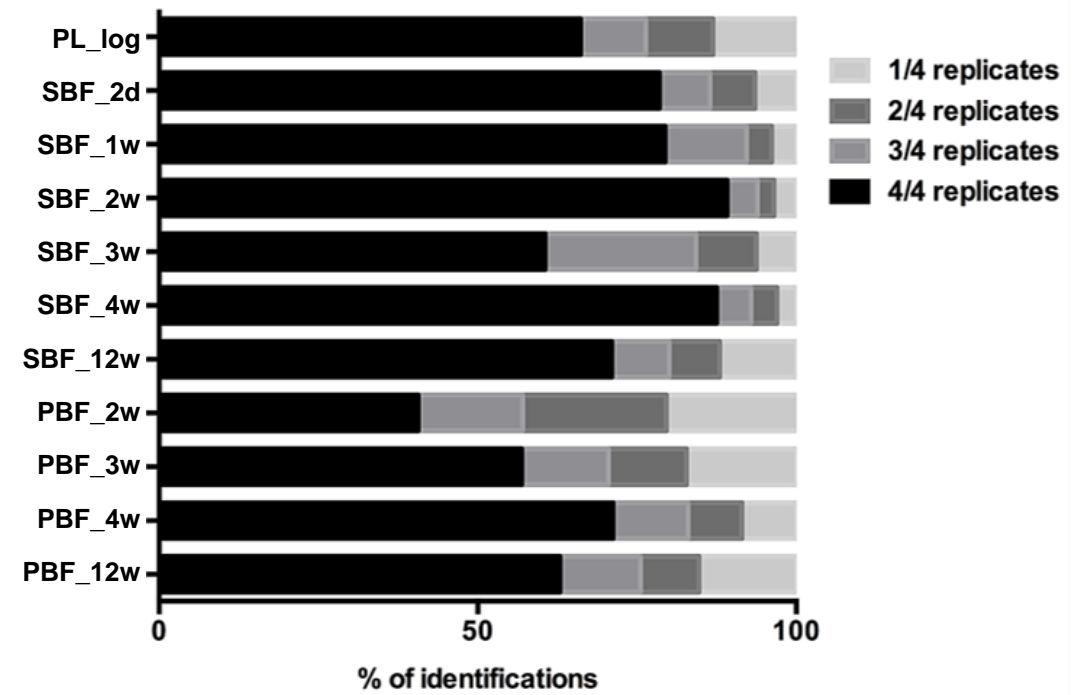

Supplement: FIG S1 [file msystems.00500-21-sf001.pdf]

A

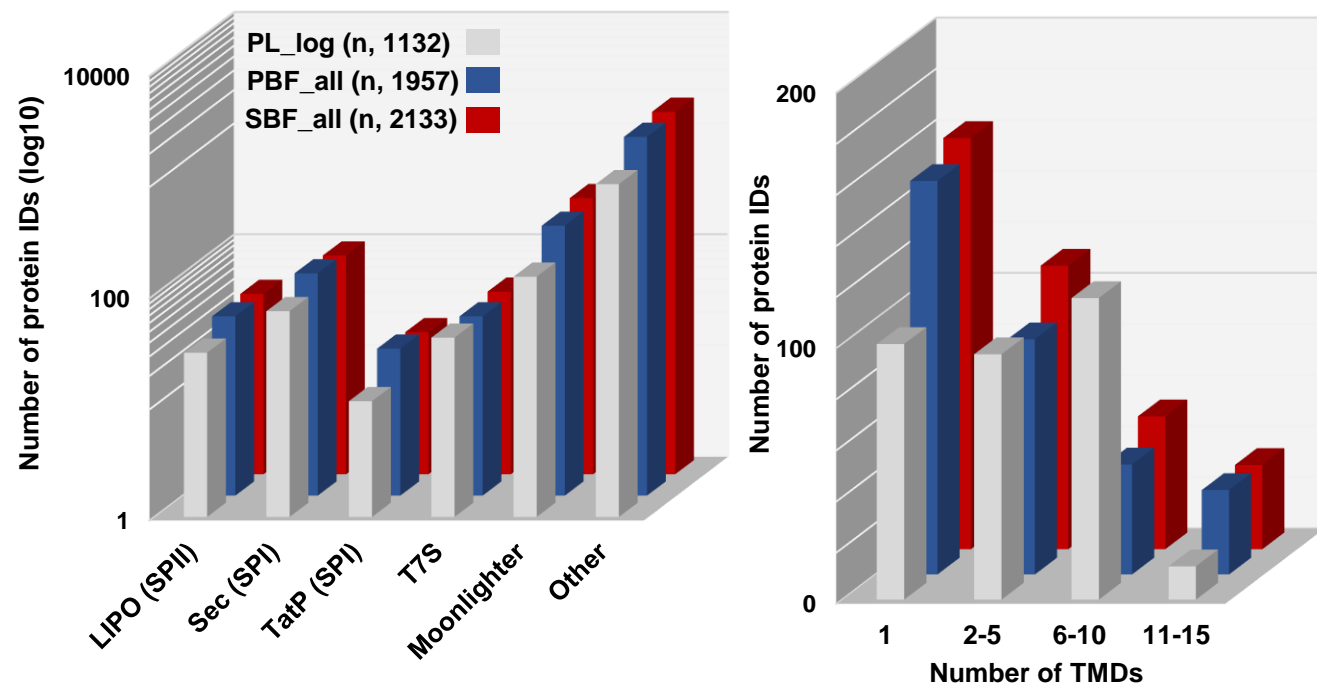

B

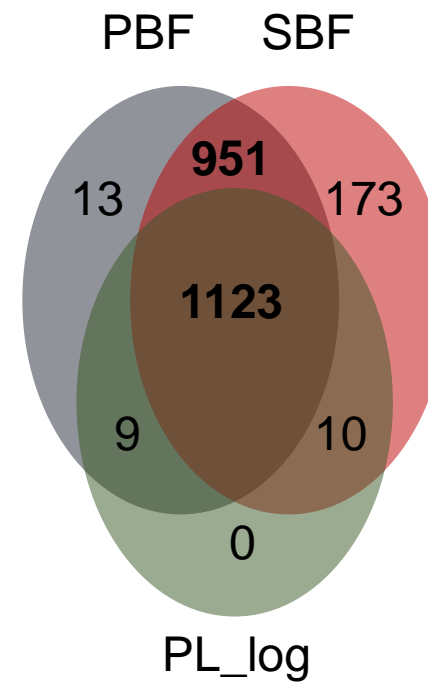

Supplement: FIG S2 [file msystems.00500-21-sf002.pdf]

A

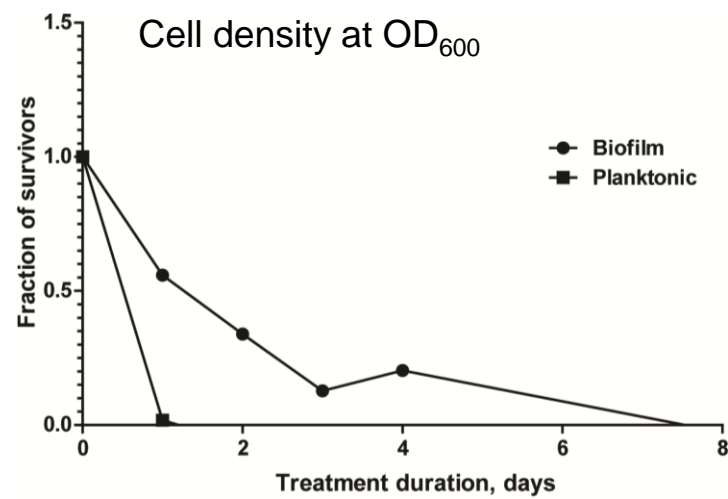

B

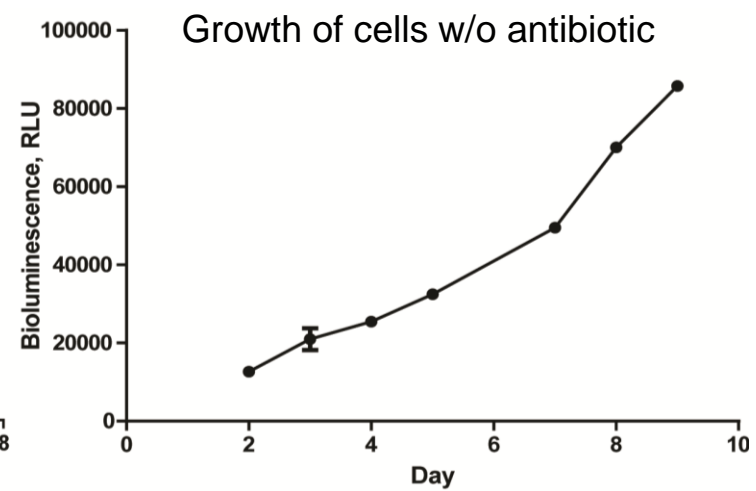

Supplement: FIG S3 [file msystems.00500-21-sf003.pdf]
