# Supplementary material for: Surface-Shaving Proteomics of Mycobacterium marinum Identifies Biofilm Subtype-Specific Changes Affecting Virulence, Tolerance, and Persistence
Source: mSystems. 2021 Jun 22;6(3):e00500-21. doi: 10.1128/mSystems.00500-21 (PMC8269238; doi:10.1128/mSystems.00500-21)
Supplement: TABLE S6 [file msystems.00500-21-st006.docx]

| **UniProtKB** | **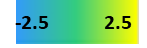Protein name** | **PL _log** | **PBF _2w** | **PBF_3w** | **PBF_4w** | **PBF_12w** | **SBF_2d** | **SBF_1w** | **SBF_2w** | **SBF_3w** | **SBF_4w** | **SBF_12w** |
| --- | --- | --- | --- | --- | --- | --- | --- | --- | --- | --- | --- | --- |
| **1. Secretion (Virulence)^(a^** | |  |  |  |  |  |  |  |  |  |  |  |
| A0A2Z5YPB5_MYCMR | ESAT-6-like protein - EsxA |  |  |  |  |  |  |  |  |  |  |  |
| A0A2D1N560_MYCMR | ESAT-6-like protein - EsxB |  |  |  |  |  |  |  |  |  |  |  |
| A0A2Z5YNV9_MYCMR | ESX-1 secretion system protein EccA1 |  |  |  |  |  |  |  |  |  |  |  |
| A0A2Z5YPK8_MYCMR | ESX-1 secretion system protein EccB1 |  |  |  |  |  |  |  |  |  |  |  |
| A0A346FAT9_MYCMR | ESX-1 secretion system protein EccCb1 |  |  |  |  |  |  |  |  |  |  |  |
| A0A2Z5YNR7_MYCMR | ESX-1 secretion system protein EspB |  |  |  |  |  |  |  |  |  |  |  |
| A0A2Z5YPA6_MYCMR | ESX-1 secretion system protein EspF |  |  |  |  |  |  |  |  |  |  |  |
| A0A2Z5YPV4_MYCMR | ESX-1 secretion system protein EspG1 |  |  |  |  |  |  |  |  |  |  |  |
| A0A2Z5YNV7_MYCMR | ESX-1 secretion system protein EspH |  |  |  |  |  |  |  |  |  |  |  |
| A0A2Z5YP05_MYCMR | ESX-1 secretion system protein EspL |  |  |  |  |  |  |  |  |  |  |  |
| A0A346EWS1_MYCMR | ESX-3 secretion system protein EccA3 |  |  |  |  |  |  |  |  |  |  |  |
| A0A2Z5Y948_MYCMR | ESX-3 secretion system protein EccD3 |  |  |  |  |  |  |  |  |  |  |  |
| A0A2Z5YF42_MYCMR | ESX-5 secretion system ATPase EccB5 |  |  |  |  |  |  |  |  |  |  |  |
| A0A346F2Y9_MYCMR | ESX-5 secretion system protein EccA5 |  |  |  |  |  |  |  |  |  |  |  |
| A0A2Z5YF84_MYCMR | ESX-5 secretion system protein EspG5 |  |  |  |  |  |  |  |  |  |  |  |
| A0A2Z5Y8K7_MYCMR | Mammalian cell entry protein Mce |  |  |  |  |  |  |  |  |  |  |  |
| A0A2Z5Y9K5_MYCMR | Heparin-binding hemagglutinin HbhA |  |  |  |  |  |  |  |  |  |  |  |
| A0A2Z5YNT8_MYCMR | Immunogenic protein MPB64/MPT64 |  |  |  |  |  |  |  |  |  |  |  |
| A0A2Z5YPS1_MYCMR | Ferritin (iron storage) - BfrB |  |  |  |  |  |  |  |  |  |  |  |
| A0A2Z5YNY0_MYCMR | Immunogenic protein PPE68 |  |  |  |  |  |  |  |  |  |  |  |
| A0A346F2Y6_MYCMR | EsX-5 secretion protein EccD |  |  |  |  |  |  |  |  |  |  |  |
| A0A2Z5Y8U8_MYCMR | Esx-5 secretion protein EccC |  |  |  |  |  |  |  |  |  |  |  |
| A0A2Z5YNR3_MYCMR | Esx-5 secretion protein EccCa |  |  |  |  |  |  |  |  |  |  |  |
| A0A2Z5YG05_MYCMR | Esx-5 secretion protein EccE |  |  |  |  |  |  |  |  |  |  |  |
| A0A2Z5YNZ0_MYCMR | Esx-5 secretion protein EccE |  |  |  |  |  |  |  |  |  |  |  |
| A0A2Z5Y8V8_MYCMR | Esx-5 secretion protein EccE |  |  |  |  |  |  |  |  |  |  |  |
| A0A346F2Y7_MYCMR | Serine protease –_MycP (type VII secretion) |  |  |  |  |  |  |  |  |  |  |  |
| A0A2Z5YBU2_MYCMR | Protein translocase subunit SecA1 |  |  |  |  |  |  |  |  |  |  |  |
| A0A2Z5YFZ0_MYCMR | Protein translocase_subunit SecA2 |  |  |  |  |  |  |  |  |  |  |  |
| A0A2Z5YDJ7_MYCMR | Protein translocase_subunit SecD |  |  |  |  |  |  |  |  |  |  |  |
| B2HSG9_MYCMM | Protein translocase subunit SecE |  |  |  |  |  |  |  |  |  |  |  |
| A0A2Z5YDT6_MYCMR | Protein export membrane protein SecF |  |  |  |  |  |  |  |  |  |  |  |
| A0A2Z5YIU9_MYCMR | Lectin - carbohydrate binding protein |  |  |  |  |  |  |  |  |  |  |  |
| A0A346F347_MYCMR | Fibronectin (Fn) binding protein Apa (antigen) |  |  |  |  |  |  |  |  |  |  |  |
| **2. Cell wall & Membrane biogenesis** | | | |  |  |  |  |  |  |  |  |  |
| A0A100I2Q3_9MYCO | Sensor-type histidine kinase PrrB |  |  |  |  |  |  |  |  |  |  |  |
| A0A124BUY4_9MYCO | Serine/threonine protein phosphatase |  |  |  |  |  |  |  |  |  |  |  |
| A0A2Z5Y7G9_MYCMR | Serine/threonine protein kinase PknA |  |  |  |  |  |  |  |  |  |  |  |
| A0A2Z5Y7G7_MYCMR | Serine/threonine protein kinase PknB |  |  |  |  |  |  |  |  |  |  |  |
| A0A2Z5YJS3_MYCMR | Adenylate cyclase |  |  |  |  |  |  |  |  |  |  |  |
| A0A346EYC2_MYCMR | Cutinase/Serine esterase |  |  |  |  |  |  |  |  |  |  |  |
| A0A117DTS6_9MYCO | Trehalase |  |  |  |  |  |  |  |  |  |  |  |
| A0A2Z5Y7F8_MYCMR | Cell wall synthesis protein CwsA |  |  |  |  |  |  |  |  |  |  |  |
| B2HGQ8_MYCMM | Cell wall synthesis protein Wag31 |  |  |  |  |  |  |  |  |  |  |  |
| B2HK80_MYCMM | Penicillin-binding protein 1A |  |  |  |  |  |  |  |  |  |  |  |
| A0A2Z5YP32_MYCMR | N-acetylmuramoyl-L-alanine amidase CwlM |  |  |  |  |  |  |  |  |  |  |  |
| A0A346F3Z2\|A0A346F3Z2_MYCMR | Phenolphthiocerol synthesis - Pks15 |  |  |  |  |  |  |  |  |  |  |  |
| A0A346F0D1\|A0A346F0D1_MYCMR | DIM/PGL methyl transferase |  |  |  |  |  |  |  |  |  |  |  |
| B2HIN1_MYCMM | Phenolphthiocerol synthesis – Pks/ PpsA |  |  |  |  |  |  |  |  |  |  |  |
| B2HIN0_MYCMM | Phenolphthiocerol synthesis – Pks/ PpsB |  |  |  |  |  |  |  |  |  |  |  |
| A0A2Z5YCW3_MYCMR | Phenolphthiocerol synthesis – Pks/ PpsD |  |  |  |  |  |  |  |  |  |  |  |
| A0A2Z5YCB0_MYCMR | Polyketide synthase PpsC |  |  |  |  |  |  |  |  |  |  |  |
| A0A2Z5YL45_MYCMR | Polyketide cyclase |  |  |  |  |  |  |  |  |  |  |  |
| A0A346F0C8_MYCMR | PDIM - phthiocerol dimycocerosate exporter MmpL7 |  |  |  |  |  |  |  |  |  |  |  |
| A0A346EWH8_MYCMR | TMM - trehalose monomycolate exporter MmpL3 |  |  |  |  |  |  |  |  |  |  |  |
| A0A2Z5YE06_MYCMR | 3-oxoacyl-[ACP] reductase FabG1 |  |  |  |  |  |  |  |  |  |  |  |
| A0A2Z5YH19_MYCMR | 3-oxoacyl-[ACP] synthase |  |  |  |  |  |  |  |  |  |  |  |
| A0A2Z5YCV1_MYCMR | Long-chain-fatty-acid-AMP ligase FadD28 (PDIM) |  |  |  |  |  |  |  |  |  |  |  |
| A0A2Z5YEA0_MYCMR | Long-chain-fatty-acid-AMP ligase FadD25 |  |  |  |  |  |  |  |  |  |  |  |
| A0A2Z5YC91_MYCMR | Long-chain-fatty-acid-AMP ligase FadD29 (PDIM) |  |  |  |  |  |  |  |  |  |  |  |
| A0A2Z5YPN8_MYCMR | Long-chain-fatty-acid-AMP ligase FadD32 |  |  |  |  |  |  |  |  |  |  |  |
| A0A2Z5YHL1_MYCMR | Long-chain-fatty-acid-CoA ligase FadD15 |  |  |  |  |  |  |  |  |  |  |  |
| A0A2Z5YL93_MYCMR | Long-chain-acyl-CoA synthetase |  |  |  |  |  |  |  |  |  |  |  |
| A0A2Z5YEH9_MYCMR | Diacylglycerol O-acyltransferase/Tgs1 (wax synthesis) |  |  |  |  |  |  |  |  |  |  |  |
| A0A2Z5YD42_MYCMR | Multifunctional mycocerosic acid synthase |  |  |  |  |  |  |  |  |  |  |  |
| A0A2Z5YNG5_MYCMR | Acyl-[ACP] desaturase DesA1 |  |  |  |  |  |  |  |  |  |  |  |
| A0A2Z5YLR2_MYCMR | Acyl-[ACP] desaturase DesA2 |  |  |  |  |  |  |  |  |  |  |  |
| A0A2Z5YNM3_MYCMR | Arabinosyltransferase A - EmbA |  |  |  |  |  |  |  |  |  |  |  |
| A0A2Z5YNT0_MYCMR | Arabinosyltransferase B - EmbB |  |  |  |  |  |  |  |  |  |  |  |
| A0A117DTZ4_9MYCO | Mycolyltransferase Ag85C (TDM synthesis), binds Fn |  |  |  |  |  |  |  |  |  |  |  |
| Q7X511_MYCMR | Mycolyltransferase Ag85A (TDM synthesis), binds Fn |  |  |  |  |  |  |  |  |  |  |  |
| A0A2Z5Y9U7_MYCMR | Cyclopropane mycolic acid synthase – PcaA/Cma2 |  |  |  |  |  |  |  |  |  |  |  |
| A0A2Z5Y9S2_MYCMR | Mycolic acid methyltransferase MmaA1 |  |  |  |  |  |  |  |  |  |  |  |
| A0A2Z5YA54_MYCMR | Methoxy mycolic acid synthase MmaA3 |  |  |  |  |  |  |  |  |  |  |  |
| A0A2Z5YA28_MYCMR | Hydroxymycolate synthase MmaA4 |  |  |  |  |  |  |  |  |  |  |  |
| A0A2Z5YP32_MYCMR | N-acetylmuramoyl-L-alanine amidase CwlM |  |  |  |  |  |  |  |  |  |  |  |
| A0A2Z5YPD0_MYCMR | Peptidoglycan biosynthesis protein MviN |  |  |  |  |  |  |  |  |  |  |  |
| A0A2Z5YHF0_MYCMR | Acyl carrier protein (ACP) - KasA |  |  |  |  |  |  |  |  |  |  |  |
| A0A2Z5YE10_MYCMR | Enoyl-[acyl-carrier-protein] reductase - InhA |  |  |  |  |  |  |  |  |  |  |  |
| A0A2Z5YMW8_MYCMR | D-alanyl-D-alanine carboxypeptidase - DacB |  |  |  |  |  |  |  |  |  |  |  |
| A0A2Z5YDQ8_MYCMR | Lipoarabinomannan carrier protein - LprG |  |  |  |  |  |  |  |  |  |  |  |
|  |  |  |  |  |  |  |  |  |  |  |  |  |
| **3. Stress response** | |  |  |  |  |  |  |  |  |  |  |  |
| A0A2Z5YB02_MYCMR | 10 kDa chaperone protein GroES, Cpn10) |  |  |  |  |  |  |  |  |  |  |  |
| A0A2Z5YAT4_MYCMR | 60 kDa chaperone protein GroEL, Cpn60) |  |  |  |  |  |  |  |  |  |  |  |
| A0A2Z5Y9I0_MYCMR | Co-chaperon GrpE (HSP70_cofactor) |  |  |  |  |  |  |  |  |  |  |  |
| A0A346EWZ4_MYCMR | Chaperone protein DnaK |  |  |  |  |  |  |  |  |  |  |  |
| A0A2Z5Y952_MYCMR | Chaperone protein ClpB |  |  |  |  |  |  |  |  |  |  |  |
| A0A2Z5YIA3_MYCMR | Chaperone protein DnaJ |  |  |  |  |  |  |  |  |  |  |  |
| A0A2Z5Y928_MYCMR | Chaperone protein DnaK_(HSP70) |  |  |  |  |  |  |  |  |  |  |  |
| A0A2Z5YI10_MYCMR | Chaperone protein HtpG |  |  |  |  |  |  |  |  |  |  |  |
| A0A100I3R0_9MYCO | Cold-shock_protein - CpsD |  |  |  |  |  |  |  |  |  |  |  |
| A0A2Z5YM49_MYCMR | Cold shock_protein - CpsA |  |  |  |  |  |  |  |  |  |  |  |
| A0A2Z5YBD5_MYCMR | Endopeptidase Lon |  |  |  |  |  |  |  |  |  |  |  |
| A0A2Z5YHP6_MYCMR | Leucyl aminopeptidase |  |  |  |  |  |  |  |  |  |  |  |
| A0A2Z5YE53_MYCMR | X-Pro dipeptidase – PepD |  |  |  |  |  |  |  |  |  |  |  |
| B2HJ41_MYCMM | ATP-dependent ClpATPase C - ClpC (chaperone) |  |  |  |  |  |  |  |  |  |  |  |
| A0A346F6C3_MYCMR | Aminopeptidase (PepN or PepC ) |  |  |  |  |  |  |  |  |  |  |  |
| A0A2Z5YJX9_MYCMR | ATP-dependent ClpATPase X – ClpX (chaperone) |  |  |  |  |  |  |  |  |  |  |  |
| A0A2Z5YJU4_MYCMR | ATP-dependent protease P - ClpP |  |  |  |  |  |  |  |  |  |  |  |
| A0A2Z5YMV3_MYCMR | ATP-dependent Zn-metalloprotease FtsH |  |  |  |  |  |  |  |  |  |  |  |
| A0A2Z5YM44_MYCMR | Oligopeptidase B – PepB |  |  |  |  |  |  |  |  |  |  |  |
| A0A346F799_MYCMR | Serine protease HtrA |  |  |  |  |  |  |  |  |  |  |  |
| A0A2Z5Y8F7_MYCMR | Serine protease PepA |  |  |  |  |  |  |  |  |  |  |  |
| 0A2Z5YNG9_MYCMR | Serine_protease_MarP (periplasmic) |  |  |  |  |  |  |  |  |  |  |  |
| A0A346F6C3_MYCMR | Aminopeptidase (PepN/PepC) |  |  |  |  |  |  |  |  |  |  |  |
| A0A2Z5YJX8_MYCMR | Beta carbonic anhydrase, 17.7 kDa |  |  |  |  |  |  |  |  |  |  |  |
| A0A2Z5YMS9_MYCMR | Beta carbonic anhydrase_βCA2 |  |  |  |  |  |  |  |  |  |  |  |
| **4. TCA/glyoxylate cycles & Carbohydrate metabolism** | |  |  |  |  |  |  |  |  |  |  |  |
| A0A2Z5YCN4_MYCMR | Acetolactate synthase |  |  |  |  |  |  |  |  |  |  |  |
| A0A2Z5Y8S5_MYCMR | Fumarate reductase_ |  |  |  |  |  |  |  |  |  |  |  |
| A0A2Z5YL78_MYCMR | Citrate synthase – CitA |  |  |  |  |  |  |  |  |  |  |  |
| A0A2Z5Y7T8_MYCMR | Isocitrate dehydrogenase ddedehydrdehydrogenase/Oxalosuccinate_decarboxylase |  |  |  |  |  |  |  |  |  |  |  |
| A0A2Z5Y9L7_MYCMR | Isocitrate lyase 1 - ICL1 |  |  |  |  |  |  |  |  |  |  |  |
| A0A2Z5YFQ2_MYCMR | Isocitrate lyase 2 – ICL2 |  |  |  |  |  |  |  |  |  |  |  |
| A0A2Z5YE47_MYCMR | Aconitate hydratase - ACN |  |  |  |  |  |  |  |  |  |  |  |
| A0A2Z5Y8W9_MYCMR | Succinate dehydrogenase - SDH |  |  |  |  |  |  |  |  |  |  |  |
| A0A2Z5YJR6_MYCMR | Malate dehydrogenase - MDH |  |  |  |  |  |  |  |  |  |  |  |
| A0A2Z5YIP1_MYCMR | 2-oxoglutarate oxidoreductase – KorB |  |  |  |  |  |  |  |  |  |  |  |
| A0A346F809_MYCMR | Enolase - ENO |  |  |  |  |  |  |  |  |  |  |  |
| A0A2Z5Y989_MYCMR | Fructose-bisphosphate aldolase - FBA |  |  |  |  |  |  |  |  |  |  |  |
| A0A2Z5YIJ8_MYCMR | Trigger factor (TF) |  |  |  |  |  |  |  |  |  |  |  |
| **5. Oxidative stress (Energy metabolism)** | |  |  |  |  |  |  |  |  |  |  |  |
| A0A2Z5Y9G3_MYCMR | Superoxide dismutase [Cu-Zn] - SodA |  |  |  |  |  |  |  |  |  |  |  |
| A0A2Z5YF87_MYCMR | Alkyl hydroperoxide reductase - AhpD |  |  |  |  |  |  |  |  |  |  |  |
| A0A2Z5YFI6_MYCMR | Alkyl hydroperoxide reductase - AhpC |  |  |  |  |  |  |  |  |  |  |  |
| A0A2Z5YFU6_MYCMR | Catalase-peroxidase (CP) - KatG |  |  |  |  |  |  |  |  |  |  |  |
| A0A2Z5YNV0_MYCMR | Thioredoxin |  |  |  |  |  |  |  |  |  |  |  |
| A0A2Z5YAP9_MYCMR | Oxidoreductase |  |  |  |  |  |  |  |  |  |  |  |
| A0A2Z5YGI0_MYCMR | Oxidoreductase |  |  |  |  |  |  |  |  |  |  |  |
| A0A346F4Z9_MYCMR | Oxidoreductase |  |  |  |  |  |  |  |  |  |  |  |
| A0A2Z5YF13_MYCMR | Oxidoreductase |  |  |  |  |  |  |  |  |  |  |  |
| A0A2Z5YMQ1_MYCMR | Oxidoreductase |  |  |  |  |  |  |  |  |  |  |  |
| A0A346F256_MYCMR | Peroxiredoxin |  |  |  |  |  |  |  |  |  |  |  |
| A0A2Z5YJS8_MYCMR | ATP synthase F1 subunit epsilon (F1F0 ATP synthase) |  |  |  |  |  |  |  |  |  |  |  |
| A0A2Z5YJL0_MYCMR | ATP synthase F1 subunit gamma (F1F0 ATP synthase) |  |  |  |  |  |  |  |  |  |  |  |
| A0A2Z5YJX5_MYCMR | ATP synthase subunit alpha (F1F0 ATP synthase) |  |  |  |  |  |  |  |  |  |  |  |
| A0A2Z5YKU5_MYCMR | ATP synthase subunit beta (F1F0 ATP synthase) |  |  |  |  |  |  |  |  |  |  |  |
| A0A346F6X0_MYCMR | ATP synthase subunit beta (F1F0 ATP synthase) |  |  |  |  |  |  |  |  |  |  |  |
| A0A2Z5YHM2_MYCMR | Cytochrome bc1 complex cytochrome_b |  |  |  |  |  |  |  |  |  |  |  |
| A0A2Z5YGS2_MYCMR | Cytochrome bc1 complex cytochrome_c |  |  |  |  |  |  |  |  |  |  |  |
| A0A2Z5YCS1_MYCMR | Cytochrome c oxidase subunit 1 |  |  |  |  |  |  |  |  |  |  |  |
| A0A346F4J4_MYCMR | Cytochrome c oxidase subunit 2 |  |  |  |  |  |  |  |  |  |  |  |

1. *Accession no. and protein names in black, proteins predicted to enter the cell membrane/cell wall/biofilm matrix via the classical secretion pathway (sec translocon or type VII secretion system); accession no. protein names in red, predicted known and new cytoplasmic moonlighters.*
